# Association of serum A20 levels with stroke-associated pneumonia, early neurological deterioration, and poor neurological prognosis following acute supratentorial intracerebral hemorrhage: a prospective cohort study

**DOI:** 10.3389/fneur.2025.1546934

**Published:** 2025-04-24

**Authors:** Chao Tang, Wei Li, Suijun Zhu, Min Zhang, Gaofeng Xiong, Yijuan Lin

**Affiliations:** ^1^Department of Neurosurgery, First People’s Hospital of Linping District, Hangzhou, China; ^2^Department of Neurosurgery, Linping Campus, The Second Affiliated Hospital of Zhejiang University School of Medicine, Hangzhou, China; ^3^Department of Critical Care Medicine, Linping Campus, The Second Affiliated Hospital of Zhejiang University School of Medicine, Hangzhou, China; ^4^Emergency Department, Linping Campus, The Second Affiliated Hospital of Zhejiang University School of Medicine, Hangzhou, China

**Keywords:** A20, intracerebral hemorrhage, outcome, severity, biomarkers

## Abstract

**Background:**

A20 is an endogenous protective protein. We quantified serum A20 levels following acute intracerebral hemorrhage (ICH) and assessed their association with the severity of illness and clinical outcomes of patients.

**Methods:**

In total, 243 patients with acute supratentorial ICH and 76 controls were included in this prospective cohort study. Serum A20 levels were measured at admission in all patients, at study entry in all controls, and on post-ICH days 1, 3, 5, 7, 10, and 14 in 76 patients. The National Institutes of Health Stroke Scale (NIHSS) scores and hematoma volume were used to estimate the severity. Stroke-associated pneumonia (SAP), early neurological deterioration (END), and post-ICH 6-month poor prognosis (modified Rankin Scale scores: 3–6) were considered as the three outcome variables of interest.

**Results:**

Patients, as opposed to controls, exhibited significantly heightened serum A20 levels from admission until 14 days following ICH, with a peak value at day 3. Serum A20 levels at all-time points after ICH, which were significantly correlated with NIHSS scores and hematoma volume, were significantly higher in patients with END, SAP, or poor prognosis than in those without the corresponding one. Serum A20 levels at admission possessed similar predictive ability of these clinical outcomes to those at other time points. Serum A20 levels at admission, along with initial NIHSS scores and hematoma volume, remained independent predictors of clinical outcomes among patients. As confirmed by numerous statistical approaches, their conjunctions comprised three prediction models: satisfactory stability, clinical validity, and discrimination efficiency.

**Conclusion:**

Serum A20 levels were significantly increased following ICH and may accurately reflect hemorrhagic severity and effectively predict END, SAP, and poor neurological prognosis, suggesting that serum A20 may be a promising prognostic biomarker for ICH.

## 1 Introduction

Spontaneous intracerebral hemorrhage (ICH) is one of the most common cerebrovascular diseases and seriously threatens human life and health (1). Etiologies of primary ICH principally are age, hypertension, diabetes mellitus, cigarette smoking, and alcohol drinking (2). The pathophysiological mechanisms of ICH occurrence mainly encompass vascular atherosclerosis and amyloidosis (3). Bleeding into the brain parenchyma activates an array of cascading molecular reactions, such as inflammation, oxidative stress, and cellular apoptosis, thereby resulting in neurological impairments (4). Clinically, the National Institutes of Health Stroke Scale (NIHSS) score and hematoma volume are widely used for severity estimation (5). The modified Rankin Scale (mRS) is an assessment tool of neurological outcomes in ICH (6). Early neurological deterioration (END) and stroke-associated pneumonia (SAP) are the two common complications of ICH and are highly connected with the poor prognosis of patients (7, 8). Therefore, the accurate prediction of END, SAP, and neurological outcomes is equally important during ICH management (9). Considering the easy availability of blood in clinical practice, blood biomarkers have gained great attention with respect to their clinical prospects in severity evaluation and outcome anticipation of ICH (10–13).

Neuroinflammation is a pivotal process among secondary brain injury subsequent to ICH (14). Nuclear factor-kappa B (NF-κB) is a key factor for driving various inflammatory pathways in the central nervous system (15). A20 is known as the tumor necrosis factor *α*-inducible protein 3, and as a central inhibitor of NF-κB, it can potently reduce the tumor necrosis factor receptor-associated factor 6/NF-κB signaling pathway (16). It is abundantly localized in neurons and astrocytes (16, 17). In response to experimental ischemic, traumatic, or hemorrhagic brain injury, A20 expressions were significantly promoted in lesion borders and held endogenous brain-protective properties (18–21). Recently, serum A20 levels were reported to be independently associated with delayed cerebral ischemia and poor prognosis following aneurysmal subarachnoid hemorrhage (22). Thus, these features could imply serum A20 as a biomarker of brain injury. Here, serum A20 levels were quantified so as to investigate their temporal alteration following ICH and their predictive effects on END, SAP, and poor prognosis of patients.

## 2 Materials and methods

### 2.1 Participants, enrollment criteria, and ethical statement

**Figure 1:**
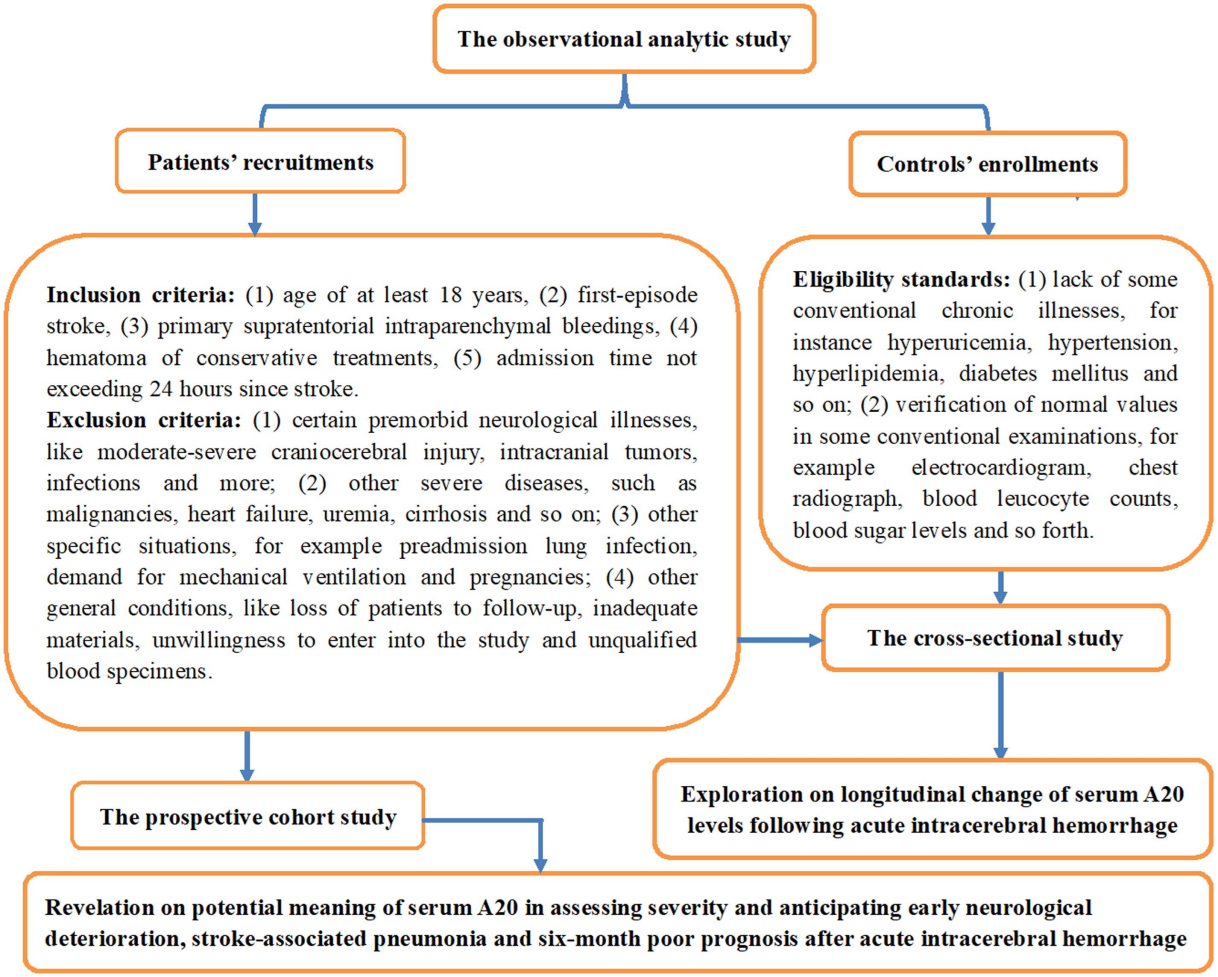
Schematic illustration of the study design and enrollment in the assessment of serum A20 as a prognostic marker for acute intracerebral hemorrhage. This clinical survey comprised two sub-studies, a cross-sectional study and a prospective cohort study, to identify serial changes in serum A20 levels post-acute intracerebral hemorrhage and their prognostic significance.

This observational analytical study was conducted at the First People’s Hospital of Linping District (Hangzhou, China) from March 2019 to July 2023, encompassing a cross-sectional study and a prospective cohort study for, respectively, determining the longitudinal alteration of serum A20 levels and their predictive ability of END, SAP, and poor prognosis after ICH. An initial assessment was done in adult patients admitted within 24 h of the first-episode primary supratentorial intraparenchymal hemorrhage for conservative treatments; and thereafter, some patients were excluded owing to certain conditions which are outlined in Figure 1. Meanwhile, a group of volunteers were enlisted as controls according to the criteria outlined in Figure 1. This study was performed in compliance with the Declaration of Helsinki and its later updates, and the study protocol was approved by the Ethics Committee at the First People’s Hospital of Linping District (approval number: LPH2019018). The patients’ lawful proxies and controls themselves were thoroughly informed of the study contents, and then they signed informed consent forms independently.

### 2.2 Collection of basic clinical information

Information regarding the age, sex, tobacco smoking habits, and alcohol consumption patterns of the patients was collected via inquiries with relatives upon arrival at the emergency department. Previously diagnosed comorbidities, such as hypertension, diabetes mellitus, chronic obstructive pulmonary disease, and ischemic heart disease, were registered. Certain premorbid medications, such as statins, anticoagulants, and antiplatelet agents, were documented. Vital signs, such as blood pressure, heart rate, and body temperature, were assessed noninvasively. To assess the neurological circumstances, the initial NIHSS score was recorded at admission (23). Clinical manifestations were noted and vomiting was observed. Dysphagia was assessed based on the results of the swallowing test. By browsing the head computerized tomography imaging, the hematoma volume was computed by referring to the volume of sphere with the application of the following formula: 0.5 × a × b × c (24). The hematomas were localized in the superficial and deep areas. Leakage of blood clots into the intraventricular or subarachnoid spaces was observed. An elevation of at least 4 points in terms of NIHSS scores or death within 24 h post admission was reported as END (25). The diagnostic criteria for SAP were formulated by a consensus group as the emergence of lower airway infections within the first 7 days post ICH (26). SAP was diagnosed in accordance with clinical and laboratory parameters of respiratory tract infection (e.g., fever, new purulent sputum, cough, bronchial breath sounds, or worsening gas exchange) and confirmed by typical chest radiographic manifestations (26). To evaluate the neurological status of each patient 6 months after acute ICH, the modified Rankin Scale (mRS) was used, with scores of 3–6 signifying the poor prognosis (27). Here, the primary study outcome was poor prognosis, and the secondary study outcomes included SAP and END.

### 2.3 Sample collection and assays

Venous blood specimens were obtained at the emergency department via antecubital venipuncture from all patients. Some patients were also willing to supply blood samples on days 1, 3, 5, 7, 10, and 14 following ICH. Subsequently, their blood samples were obtained by using the means described above. For the controls, venous blood was extracted at the commencement of the study. Blood specimens were placed into 5 mL gel-embedded plastic vacuum collection vessels. The coagulated blood samples were spun at 2,000 × g for 10 min. Subsequently, the supernatants were aspirated and promptly transferred to Eppendorf tubes for further storage in a low-temperature freezer at −80°C, awaiting eventual measurements. To prevent protein breakdown, serum samples were thawed for biomarker measurements within a quarter of preservation. An enzyme-linked immunosorbent assay kit for detecting serum A20 levels was purchased from Shanghai Jianglai Biotechnology Co., Ltd. (Shanghai, China; Catalogue no. JL48812). The detection range varied from 30 to 5,000 pg./mL, with both intra- and inter-assay coefficients of variation <10%. According to the manufacturer’s protocol, each sample was detected twice by an identical skilled operator who was blinded to the clinical information.

### 2.4 Statistical analysis

All data were analyzed using the SPSS software (version 23.0; SPSS Inc., Chicago, IL, United States). Categorical variables were presented as counts (percentages). Normality tests for continuous variables were performed using the Kolmogorov–Smirnov test. Normally distributed continuous variables were presented as means (standard deviations, SDs), and non-normally distributed variables were reported as medians (percentiles 25th–75th). The chi-square test, Fisher’s exact test, independent-sample Student’s *t*-test, and the Mann–Whitney U test were used for data comparison between the two groups, as deemed applicable. The Kruskal–Wallis test was performed for data comparison among several groups. Three outcome variables of interest existed: SAP, END, and 6-month poor prognosis following ICH. Independent predictors of each outcome variable were identified by integrating all significantly distinct variables in the univariate analyses into binary multifactorial logistic regression models. Bivariate correlation assessments were performed using the Spearman’s test. Nomograms, calibration curves, and decision curves were created using the R 3.5.1 software.^1^ A receiver operating characteristic (ROC) curve analysis was performed using MedCalc 20 (MedCalc Software, Ltd., Ostend, Belgium), with areas under the ROC curve being compared using the Z test. The ROC curves were plotted using GraphPad Prism 7.01 (GraphPad Software, Inc., San Diego, CA, United States). In this study, all patients consented for blood collection at admission and some of them also voluntarily continued to supply blood samples at other time points, including days 1, 3, 5, 7, 10, and 14 post ICH. The univariate analysis was done only for assessing the data of those patients consenting for blood sampling at multiple time points. Univariate and multivariate analyses were sequentially performed for analyzing the data of all patients. A two-sided *p*< 0.05 was considered statistically significant.

## 3 Results

### 3.1 Study population

As required by the enrollment criteria (shown in Figure 1), 312 patients with acute ICH underwent initial screening, and the removal of 69 patients from this cohort led to the retention of 243 patients for the final investigation. Alternatively, apart from the blood sample collection at admission, 76 patients voluntarily continued to supply blood samples at other time points, including days 1, 3, 5, 7, 10, and 14 post ICH. As shown in Table 1, all 243 patients were comparable to the 76 patients in terms of baseline demographical, clinical, radiological, and biochemical data (all *p* > 0.05). A group of 76 controls, 40 being males, 28 and 27 being tobacco smokers and alcohol drinkers, respectively, aged between 44 and 77 years, with mean and SD values of 57.4 and 9.5 years, respectively, were included in the study. From a statistical perspective, negligible disparities were observed in the mean age, sex ratio, and proportion of smokers and drinkers between the 76 controls and 76 patients (all *p* > 0.05).

**Table 1:** Distinction of baseline parameters between all patients and those consenting for blood collection at multiple time points after acute intracerebral hemorrhage.

Variables	All patients	Partial patients	*p*-values
Age ≥ 65 years	84 (34.6%)	25 (32.9%)	0.788
Gender (male/female)	137/106	39/37	0.439
Cigarette consumption	84 (34.6%)	24 (31.6%)	0.631
Alcohol consumption	83 (34.2%)	23 (30.3%)	0.529
Hypertension	162 (66.7%)	55 (72.4%)	0.352
Diabetes mellitus	60 (24.7%)	21 (27.6%)	0.607
Dyslipidemia	85 (35.0%)	27 (35.5%)	0.931
COPD	10 (4.1%)	3 (3.9%)	0.948
IHD	18 (7.4%)	6 (7.9%)	0.888
Hyperuricemia	27 (11.1%)	10 (13.2%)	0.627
Statin use	64 (26.3%)	16 (21.1%)	0.354
Anticoagulant use	16 (6.6%)	5 (6.6%)	0.999
Antiplatelet use	26 (10.7%)	11 (14.5%)	0.370
Admission time (h)	8.3 (5.3–13.6)	8.3 (4.8–12.3)	0.722
Sampling time (h)	9.3 (6.3–14.8)	9.3 (5.8–13.3)	0.658
Dysphasia	53 (21.8%)	19 (25.0%)	0.562
Vomiting	57 (23.5%)	21 (27.6%)	0.460
SAP (mmHg)	141.9 ± 23.7	142.8 ± 24.7	0.695
DAP (mmHg)	87.1 ± 9.6	87.2 ± 9.4	0.870
Superficial hematoma	67 (27.6%)	22 (28.9%)	0.816
IVH	36 (14.8%)	14 (18.4%)	0.450
SAH	14 (5.8%)	4 (5.3%)	0.870
NIHSS scores	12 (8–14)	12 (8–14)	0.801
Hematoma volume (mL)	11 (9–19)	12 (9–19)	0.729
Blood WBC count (×10^9^/L)	6.7 (4.7–9.2)	6.7 (4.7–9.7)	0.637
Blood glucose levels (mmol/L)	8.8 (7.0–11.7)	9.0 (7.0–11.1)	0.878
Serum A20 levels (pg/mL)	140.0 (78.3–291.0)	188.7 (79.2–316.2)	0.460

Variables were summarized in the form of count (percentage), mean ± standard deviation, or median (upper-lower quartiles). The chi-square test, Fisher’s exact test, Student’s 𝑡-test, and the Mann–Whitney test were utilized for data comparison. NIHSS, National Institutes of Health Stroke Scale; COPD, chronic obstructive pulmonary disease; IVH, intraventricular hemorrhage; SAH, subarachnoid hemorrhage; IHD, ischemic heart disease; WBC, white blood cell; SAP, systolic arterial pressure; DAP, diastolic arterial pressure.

### 3.2 Dynamic variation of serum A20 levels and their association with ICH severity

**Figure 2:**
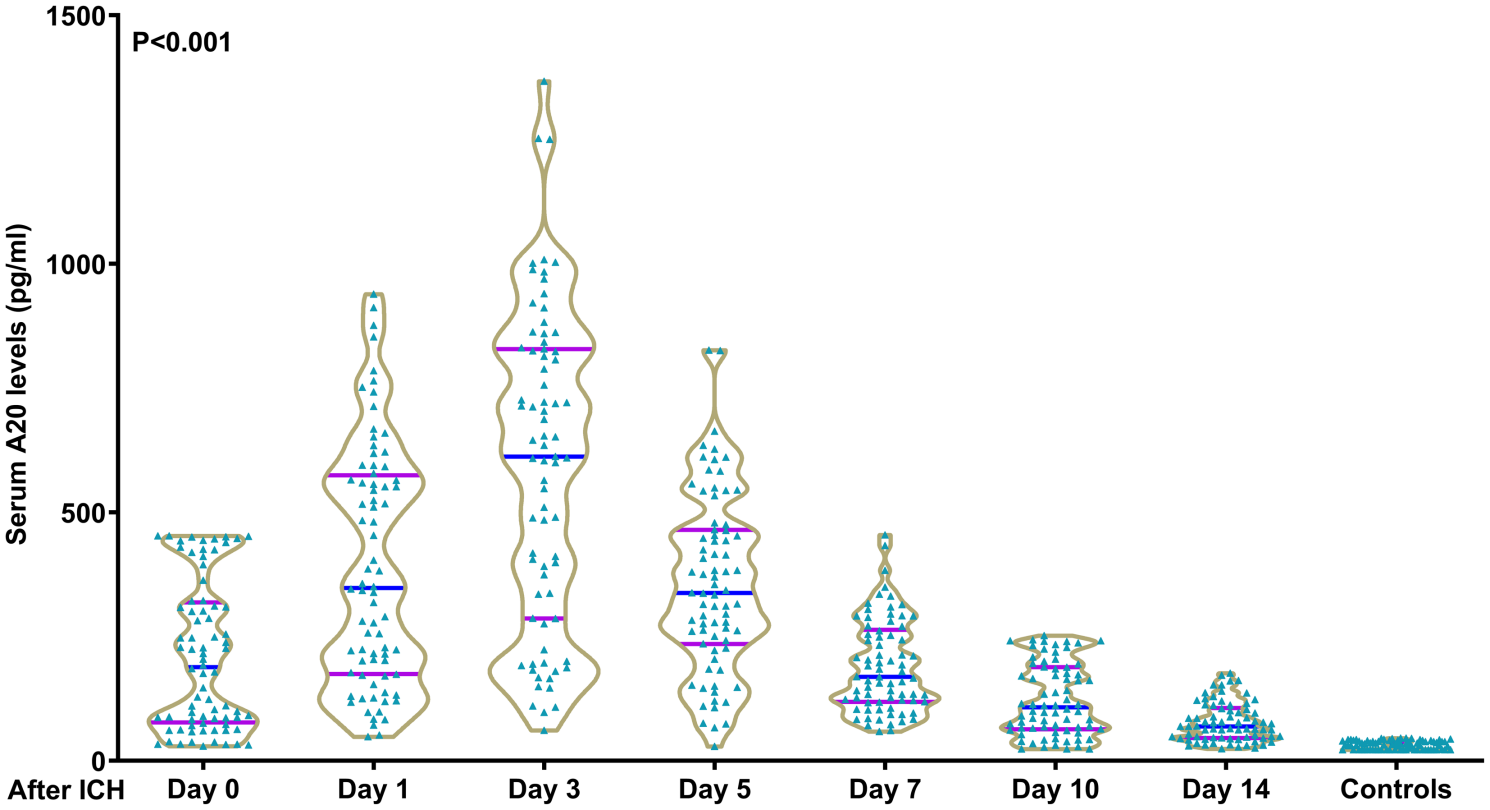
Time course of serum A20 levels in 76 patients with acute intracerebral hemorrhage. Serum A20 levels were significantly elevated at admission in patients with acute intracerebral hemorrhage, peaked on day 3, and remained significantly higher than those in controls until day 14 after stroke onset (*p* < 0.001). ICH, intracerebral hemorrhage.

In contrast to all 243 patients, 76 patients consenting for blood sample collection at multiple time points had similar serum A20 levels (*p* > 0.05) (Table 1). Among the 76 patients, serum A20 levels increased promptly after acute ICH, peaked at day 3, and significantly declined until day 14 post ICH, and were markedly higher during the 14 days than those of the 76 controls (*p* < 0.001) (Figure 2). The serum A20 levels of these 76 patients were positively correlated with the baseline NIHSS scores and hematoma volume at all seven time points, namely, from admission to day 14 following acute ICH (all *p* < 0.05) (Table 2). Consistently, serum A20 levels at admission in all 243 patients increased substantially with increasing NIHSS scores (*p* < 0.001) (Supplementary Figure 1), and a strong positive correlation was observed with hematoma volume (*p* < 0.001) (Supplementary Figure 2).

**Table 2:** Association between serum A20 levels, National Institutes of Health Stroke Scale scores, and hematoma volume in patients consenting for blood collection at multiple time points following acute intracerebral hemorrhage.

Time point	NIHSS scores		Hematoma volume	
	ρ	*P*-values	ρ	*P*-values
Day 0	0.698	<0.001	0.599	<0.001
Day 1	0.649	<0.001	0.591	<0.001
Day 3	0.640	<0.001	0.695	<0.001
Day 5	0.613	<0.001	0.587	<0.001
Day 7	0.609	<0.001	0.628	<0.001
Day 10	0.297	0.010	0.414	<0.001
Day 14	0.248	0.033	0.416	<0.001

Bivariate correlative assessment was completed by using the Spearman test. NIHSS, National Institutes of Health Stroke Scale.

### 3.3 Serum A20 levels associated with post-ICH 6-month poor prognosis

**Figure 3:**
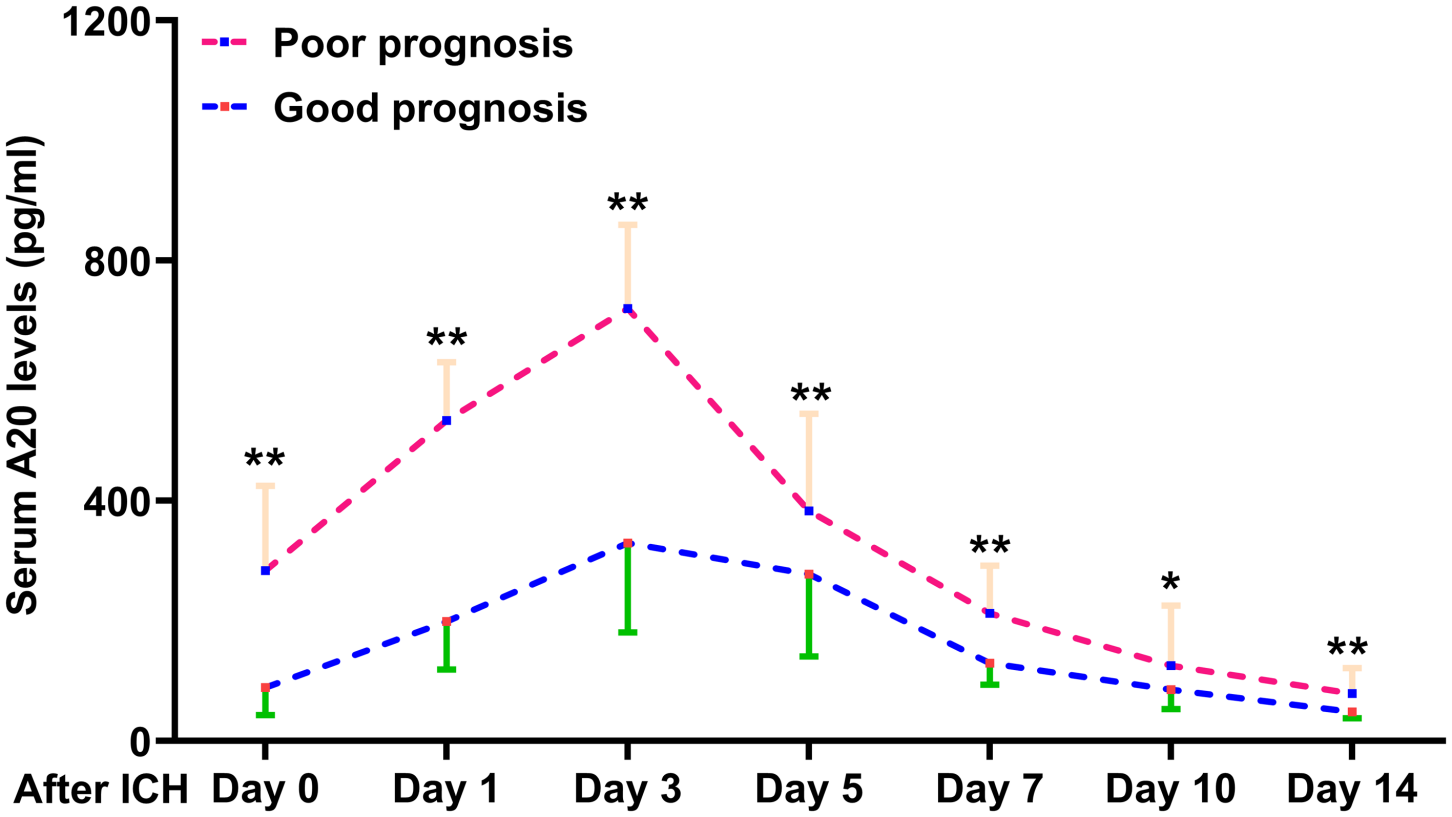
Distinction of serum A20 levels across poor prognosis at 6 months after intracerebral hemorrhage in 76 patients. Individuals with a 6-month poor prognosis had significantly higher serum A20 levels at admission and on days 1, 3, 5, 7, 10, and 14 following acute intracerebral hemorrhage than those with a good prognosis (^*^p < 0.05, ^**^p < 0.01). ICH, intracerebral hemorrhage.

**Figure 4:**
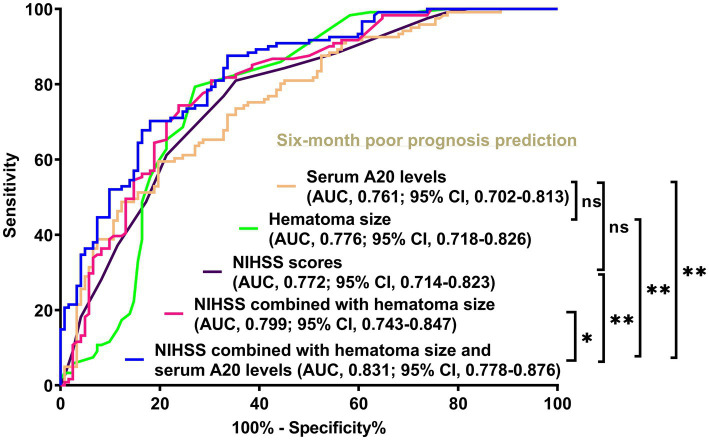
Disparities in discrimination abilities of serum A20 levels, the combination model, and other variables on the likelihood of poor prognosis at 6 months following acute intracerebral hemorrhage among all 243 patients. Serum A20 levels had an area under the receiver operating characteristic curve analogous to the National Institutes of Health Stroke Scale scores and hematoma volume (both *p* > 0.05). Regarding predictive ability, serum A20 levels combined with National Institutes of Health Stroke Scale scores and hematoma volume outperformed each of them (^*^p < 0.05, ^**^p < 0.01) in this cohort of 243 patients with acute intracerebral hemorrhage. AUC, area under the curve; 95% CI, 95% confidence interval; ns, non-significant; NIHSS, National Institutes of Health Stroke Scale.

Of the 76 patients with ICH, serum A20 levels at all seven time points were significantly higher in patients with a poor prognosis than in those without ICH (all *p* < 0.05; Figure 3). Using the depiction in Table 3, the area under the ROC curve of serum A20 levels at admission exhibited statistical resemblance to those at the remaining six time points (all *p* > 0.05). With the sample size extended to 243 patients, including 121 patients with poor prognosis, serum A20 levels exhibited effective discrimination of patients at risk of 6-month poor prognosis and predicted the outcome with a maximum Youden index of 0.398 using the optimal criterion at 208.7 pg./mL (Supplementary Figure 3). In addition, proportions of age ≥ 65 years, diabetes mellitus, dysphasia, vomiting, extension of bleeding into the ventricular system, NIHSS score, hematoma volume, blood glucose levels, and serum A20 levels substantially differed between patients who developed a poor prognosis and those who did not, among all 243 patients with acute ICH (all *p* < 0.05) (Table 4). By incorporating these nine factors of significant distinction on the univariate analysis into a multivariate model, NIHSS scores [odds ratio (OR), 1.192; 95% confidence interval (CI), 1.059–1.342; *p* = 0.001], hematoma volume (OR, 1.064; 95% CI, 1.017–1.112; *p* = 0.004), and serum A20 levels (OR, 1.004; 95% CI, 1.001–1.007; *p* = 0.009) remained independently associated with poor prognosis at 6 months following ICH. The three aforementioned independent predictors were integrated to develop a combination model of nomogram description for predicting poor prognosis in ICH (Supplementary Figure 4). The model exhibited a high consistency in prognostic prediction (Supplementary Figure 5) and satisfactory clinical validity (Supplementary Figure 6). Using ROC curve analysis, the predictive value of the serum A20 level was equivalent to those of hematoma volume and NIHSS scores (both *p* > 0.05) (Figure 4), and the predictive capability of its consolidation with hematoma volume and NIHSS scores substantially outperformed those of serum A20 levels, NIHSS scores, hematoma volume, and the combination of NIHSS scores and hematoma volume (all *p* < 0.05) (Figure 4).

**Table 3:** Differences in terms of areas under receiver operating characteristic curve of serum A20 levels at several time points among those patients allowing for blood collection at various time points following acute intracerebral hemorrhage.

Outcome variables	Time points	AUC (95% CI)	*P*-values
Poor prognosis	Day 0	0.761 (0.648–0.852)	Reference
	Day 1	0.757 (0.643–0.849)	0.897
	Day 3	0.768 (0.656–0.858)	0.869
	Day 5	0.711 (0.594–0.811)	0.369
	Day 7	0.750 (0.636–0.843)	0.855
	Day 10	0.659 (0.539–0.765)	0.161
	Day 14	0.732 (0.617–0.829)	0.696
SAP	Day 0	0.765 (0.653–0.856)	Reference
	Day 1	0.748 (0.633–0.841)	0.626
	Day 3	0.758 (0.645–0.850)	0.873
	Day 5	0.706 (0.589–0.806)	0.315
	Day 7	0.727 (0.611–0.824)	0.509
	Day 10	0.701 (0.583–0.802)	0.428
	Day 14	0.686 (0.568–0.789)	0.320
END	Day 0	0.772 (0.660–0.862)	Reference
	Day 1	0.762 (0.648–0.853)	0.778
	Day 3	0.758 (0.644–0.850)	0.775
	Day 5	0.719 (0.603–0.818)	0.399
	Day 7	0.764 (0.651–0.855)	0.892
	Day 10	0.704 (0.587–0.805)	0.384
	Day 14	0.655 (0.535–0.761)	0.112

Intergroup comparisons were achieved by applying the *Z*-test. AUC, area under curve; 95% CI, 95% confidence interval; SAP, stroke-associated pneumonia; END, early neurological deterioration.

**Table 4:** Factors associated with 6-month poor prognosis after acute intracerebral hemorrhage.

	Poor prognosis	Good prognosis	*P*-values
Age ≥ 65 years	51 (42.1%)	33 (27.0%)	0.013
Gender (male/female)	67/54	70/52	0.753
Cigarette consumption	46 (38.0%)	38 (31.1%)	0.260
Alcohol consumption	40 (33.1%)	43 (35.2%)	0.719
Hypertension	80 (66.1%)	82 (66.2%)	0.856
Diabetes mellitus	37 (30.6%)	23 (18.9%)	0.034
Dyslipidemia	47 (38.8%)	38 (31.1%)	0.209
COPD	6 (5.0%)	4 (3.3%)	0.510
IHD	11 (9.1%)	7 (5.7%)	0.318
Hyperuricemia	14 (11.6%)	13 (10.7%)	0.821
Statin use	30 (24.8%)	34 (27.9%)	0.586
Anticoagulant use	6 (5.0%)	10 (8.2%)	0.309
Antiplatelet use	15 (12.4%)	11 (9.0%)	0.394
Admission time (h)	8.3 (4.8–12.3)	8.8 (5.3–16.3)	0.064
Sampling time (h)	9.3 (5.8–13.3)	10.3 (6.3–17.3)	0.066
Dysphasia	33 (27.3%)	20 (16.4%)	0.040
Vomiting	35 (28.9%)	22 (18.0%)	0.045
SAP (mmHg)	142.1 ± 25.0	141.7 ± 22.5	0.912
DAP (mmHg)	87.0 ± 10.3	87.1 ± 8.9	0.947
Superficial hematoma	38 (31.4%)	29 (23.8%)	0.183
IVH	27 (22.3%)	9 (7.4%)	0.001
SAH	9 (7.4%)	5 (4.1%)	0.264
NIHSS scores	13 (11–16)	10 (8–13)	<0.001
Hematoma volume (mL)	16 (11–20)	9 (6–11)	<0.001
Blood WBC count (×10^9^/L)	7.2 (4.6–9.6)	6.3 (4.8–8.3)	0.343
Blood glucose levels (mmol/L)	9.5 (7.0–13.7)	8.5 (7.0–10.3)	0.001
Serum A20 levels (pg/mL)	228.2 (115.9–388.3)	97.1 (61.4–193.2)	<0.001

Data were presented as count (percentage), mean ± standard deviation, or median (upper-lower quartiles). The chi-square test, Fisher’s exact test, Student’s 𝑡-test, and the Mann–Whitney test were used for intergroup comparison. NIHSS, National Institutes of Health Stroke Scale; COPD, chronic obstructive pulmonary disease; IVH, intraventricular hemorrhage; SAH, subarachnoid hemorrhage; IHD, ischemic heart disease; WBC, white blood cell; SAP, systolic arterial pressure; DAP, diastolic arterial pressure.

### 3.4 Association of serum A20 levels with SAP after ICH

**Figure 5:**
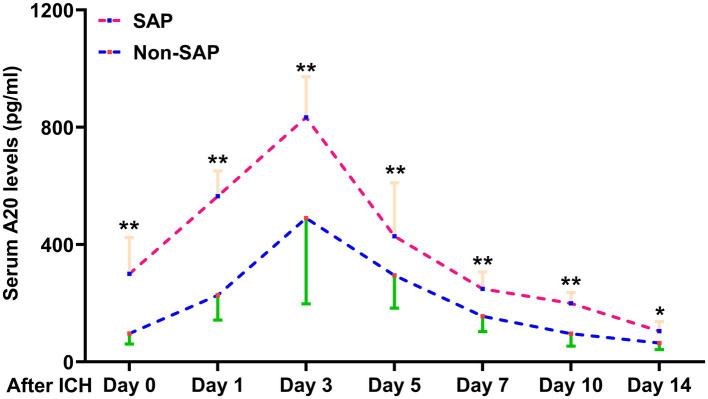
Difference in serum A20 levels across 76 patients with stroke-associated pneumonia following intracerebral hemorrhage. Patients who developed stroke-associated pneumonia, than those who did not, had significantly higher serum A20 levels at admission and on days 1, 3, 5, 7, 10, and 14 post-acute intracerebral hemorrhage among 76 patients (^*^p < 0.05, ^**^p < 0.01). ICH, intracerebral hemorrhage; SAP, stroke-associated pneumonia.

**Figure 6:**
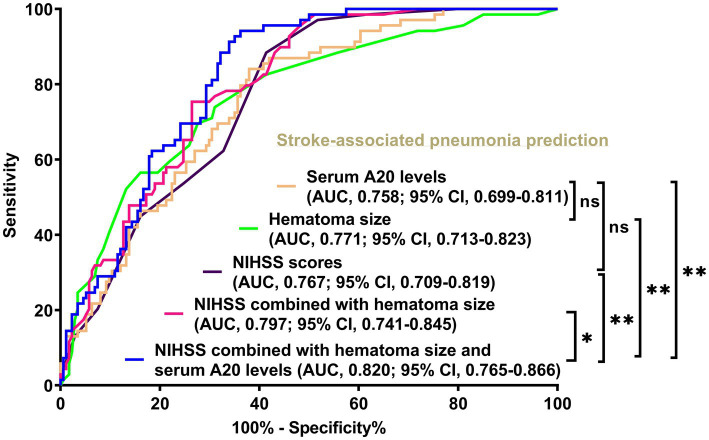
Comparison of discrimination values between serum A20 levels, combination model, and other variables on the likelihood of stroke-associated pneumonia post-acute intracerebral hemorrhage among all 243 patients. Serum A20 levels occupied the area under the receiver operating characteristic curve, similar to the National Institutes of Health Stroke Scale scores and hematoma volume (both *p* > 0.05). Regarding predictive ability, serum A20 levels combined with the National Institutes of Health Stroke Scale scores and hematoma volume surpassed each individually (^*^p < 0.05, ^**^p < 0.01) among all 243 patients with acute intracerebral hemorrhage. AUC, area under the curve; 95% CI, 95% confidence interval; ns, non-significant; NIHSS, National Institutes of Health Stroke Scale.

Of the 76 patients with acute ICH, those with SAP had significantly heightened serum A20 levels at all seven predetermined time points compared to those without SAP (all *p* < 0.05) (Figure 5). As shown in Table 3, serum A20 levels at admission exhibited no significant difference in the areas under the ROC curve compared to those at the remaining six time points in the 76 patients (all *p* > 0.05). Among all 243 patients, including 69 with SAP, SAP was effectively predicted using serum A20 levels, and an optimal threshold of 137.2 pg./mL forecast SAP with a maximum Youden index of 0.462 (Supplementary Figure 7). In addition, of all 243 patients after acute ICH, subjects experiencing SAP, exhibited substantially higher percentages of dysphagia, vomiting, and intraventricular extension of hematoma, as well as markedly increased NIHSS scores, hematoma volume, blood glucose levels, and serum A20 levels, than those without SAP development (all *p* < 0.05) (Table 5). Incorporation of the seven aforementioned variables of substantial disparity in the univariate analysis into the multivariate model revealed that NIHSS scores (OR, 1.150; 95% CI, 1.015–1.304; *p* = 0.028), hematoma volume (OR, 1.067; 95% CI, 1.014–1.123; *p* = 0.012), and serum A20 levels (OR, 1.003; 95% CI, 1.001–1.006; *p* = 0.033) were of independent association with SAP after acute ICH. A joint model encompassing the aforementioned independent variables was pictorially delineated via a nomogram for utilization in SAP prediction following ICH (Supplementary Figure 8). The model was stable in forecasting SAP (Supplementary Figure 9) with acceptable clinical benefits (Supplementary Figure 10). In terms of the area under the ROC curve, the risk-discriminating capacity of serum A20 levels resembled that of hematoma volume and NIHSS scores (both *p* > 0.05) (Figure 6), and a significantly higher predictive ability was observed for the combination model compared to serum A20 levels, NIHSS scores, hematoma volume, and the combination of NIHSS scores and hematoma volume (all *p* < 0.05) (Figure 6).

**Table 5:** Factors associated with stroke-associated pneumonia after acute intracerebral hemorrhage.

	SAP	Non-SAP	*P*-values
Age ≥ 65 years	27 (39.1%)	57 (32.8%)	0.346
Gender (male/female)	44/25	93/81	0.144
Cigarette consumption	30 (43.5%)	54 (31.0%)	0.066
Alcohol consumption	30 (43.5%)	53 (30.5%)	0.054
Hypertension	48 (69.6%)	114 (65.5%)	0.645
Diabetes mellitus	22 (31.9%)	38 (21.8%)	0.102
Dyslipidemia	22 (31.9%)	63 (36.2%)	0.524
COPD	4 (5.8%)	6 (3.4%)	0.406
IHD	4 (5.8%)	14 (8.0%)	0.546
Hyperuricemia	8 (11.6%)	19 (10.9%)	0.880
Statin use	18 (26.1%)	46 (26.4%)	0.955
Anticoagulant use	6 (8.7%)	10 (5.7%)	0.403
Antiplatelet use	9 (13.0%)	17 (9.8%)	0.457
Admission time (h)	9.3 (5.8–16.8)	8.3 (4.8–12.3)	0.089
Sampling time (h)	10.3 (7.3–18.3)	9.3 (5.8–13.8)	0.088
Dysphasia	23 (33.3%)	30 (17.2%)	0.006
Vomiting	23 (33.3%)	34 (19.5%)	0.022
SAP (mmHg)	143.2 ± 26.0	141.4 ± 22.8	0.594
DAP (mmHg)	88.0 ± 9.1	86.7 ± 9.8	0.368
Superficial hematoma	13 (18.8%)	54 (31.0%)	0.055
IVH	18 (26.1%)	18 (10.3%)	0.002
SAH	2 (2.9%)	12 (6.9%)	0.228
NIHSS scores	13 (10–16)	10 (7–13)	<0.001
Hematoma volume (mL)	20 (12–25)	10 (8–16)	<0.001
Blood WBC count (×10^9^/L)	7.3 (4.6–9.8)	6.4 (4.7–8.9)	0.211
Blood glucose levels (mmol/L)	10.6 (7.3–13.8)	8.7 (6.9–10.9)	0.045
Serum A20 levels (pg/mL)	249.1 (175.7–394.7)	105.6 (70.3–227.2)	<0.001

Data were presented as count (percentage), mean ± standard deviation, or median (upper-lower quartiles). The chi-square test, Fisher’s exact test, Student’s 𝑡-test, and the Mann–Whitney test were used for intergroup comparison. NIHSS, National Institutes of Health Stroke Scale; COPD, chronic obstructive pulmonary disease; IVH, intraventricular hemorrhage; SAH, subarachnoid hemorrhage; IHD, ischemic heart disease; WBC, white blood cell; SAP, systolic arterial pressure; DAP, diastolic arterial pressure; SAP, stroke-associated pneumonia.

### 3.5 Association of serum A20 levels with END following ICH

**Figure 7:**
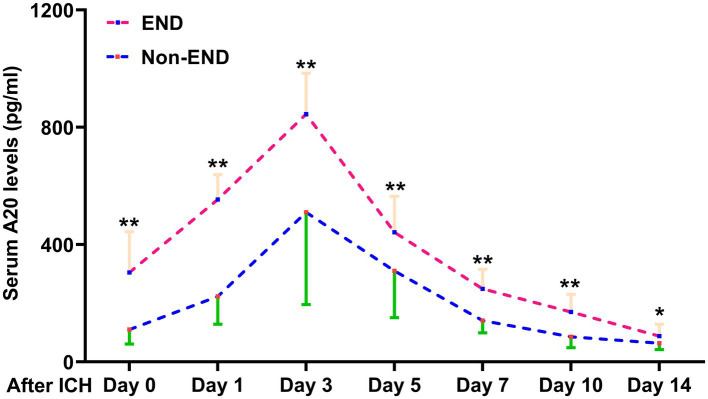
Change in serum A20 levels during early neurological deterioration in 76 patients with acute intracerebral hemorrhage. Patients with early neurological deterioration than those without such an adverse event exhibited significantly higher serum A20 levels at admission and on days 1, 3, 5, 7, 10, and 14 after acute intracerebral hemorrhage (^*^p < 0.05, ^**^p < 0.01). ICH, intracerebral hemorrhage; END, early neurological deterioration.

**Figure 8:**
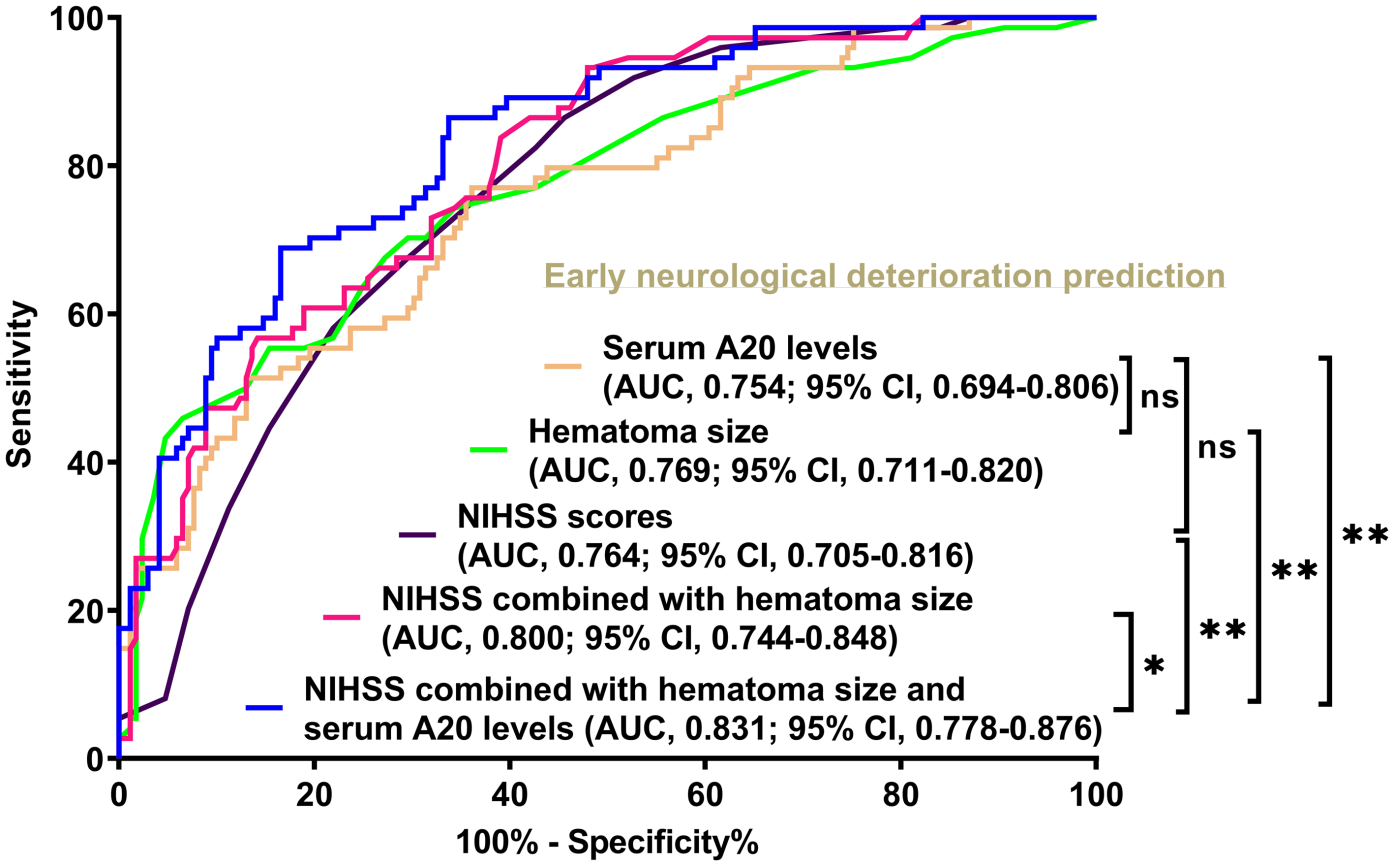
Differences in discrimination performances of serum A20 levels, the joint model, and other parameters on the likelihood of early neurological deterioration following acute intracerebral hemorrhage among all 243 patients. When serum A20 levels were compared with National Institutes of Health Stroke Scale scores and hematoma volume, the areas under the receiver operating characteristic curve were not significantly different (both *p* > 0.05). Regarding predictive capability, serum A20 levels combined with the National Institutes of Health Stroke Scale scores and hematoma volume exceeded each individually (^*^p < 0.05, ^**^p < 0.01) in all 243 patients with acute intracerebral hemorrhage. AUC, area under the curve; 95% CI, 95% confidence interval; ns, non-significant; NIHSS, National Institutes of Health Stroke Scale.

Of the 76 patients following acute ICH, serum A20 levels at all seven prespecified time points were markedly elevated in individuals with END than in those without it (all *p* < 0.05) (Figure 7). As outlined in Table 3, in contrast to the serum A20 levels at admission, the areas under the ROC curve were not significantly higher in those at the other six time points among this group of patients (all *p* > 0.05). Totally, 74 of 243 patients with ICH experienced END. Serum A20 levels were strongly predictive of END, and the levels >144.9 pg./mL prognosticated END with moderate-to-high sensitivity and specificity using the Youden method (Supplementary Figure 11). Of all 243 patients following acute ICH, patients with END, in contrast to the others, exhibited increased likelihood of antiplatelet use, anticoagulant use, and intraventricular enlargement of hematoma, as well as a profound increase in the NIHSS score, hematoma volume, blood glucose levels, and serum A20 levels (all *p* < 0.05) (Table 6). Seven significantly different parameters were entered into the multivariate model, and NIHSS scores (OR, 1.170; 95% CI, 1.048–1.306; *p* = 0.011), hematoma volume (OR, 1.087; 95% CI, 1.031–1.145; *p* = 0.019), and serum A20 levels (OR, 1.004; 95% CI, 1.001–1.008; *p* = 0.034) emerged as independent predictors of END development. A merged model in which these independent factors were entered was graphically described using a nomogram to predict END following ICH (Supplementary Figure 12). The model was operated with high stability (Supplementary Figure 13) and was demonstrated to have good clinical efficacy (Supplementary Figure 14). In light of the ROC curve, serum A20 levels exhibited analogous predictive power to hematoma volume and NIHSS scores (both *p* > 0.05) (Figure 8), and the combination model, relative to serum A20 levels, NIHSS scores, hematoma volume, and the combination of NIHSS scores with hematoma volume, possessed notably elevated predictive values (all *p* < 0.05) (Figure 8).

**Table 6:** Factors associated with early neurological deterioration after acute intracerebral hemorrhage.

	END	Non-END	*P*-values
Age ≥ 65 years	27 (36.5%)	57 (33.7%)	0.677
Gender (male/female)	46/28	91/78	0.229
Cigarette consumption	29 (39.2%)	55 (32.5%)	0.316
Alcohol consumption	30 (40.5%)	53 (31.4%)	0.165
Hypertension	52 (70.3%)	110 (65.1%)	0.430
Diabetes mellitus	23 (31.1%)	37 (21.9%)	0.126
Dyslipidemia	27 (36.5%)	58 (34.3%)	0.744
COPD	2 (2.7%)	8 (4.7%)	0.463
IHD	3 (4.1%)	15 (8.9%)	0.187
Hyperuricemia	7 (9.5%)	20 (11.8%)	0.588
Statin use	20 (27.0%)	44 (26.0%)	0.872
Anticoagulant use	9 (12.2%)	7 (4.1%)	0.020
Antiplatelet use	13 (17.6%)	13 (7.7%)	0.022
Admission time (h)	9.3 (6.3–15.3)	8.3 (4.8–13.3)	0.094
Sampling time (h)	10.3 (7.8–17.3)	9.3 (5.8–14.3)	0.067
Dysphasia	21 (28.4%)	32 (18.9%)	0.101
Vomiting	23 (31.1%)	34 (20.1%)	0.063
SAP (mmHg)	143.5 ± 25.4	141.2 ± 23.0	0.488
DAP (mmHg)	88.1 ± 9.2	86.6 ± 9.7	0.255
Superficial hematoma	20 (27.0%)	47 (27.8%)	0.900
IVH	19 (25.7%)	17 (10.1%)	0.002
SAH	6 (8.1%)	8 (4.7%)	0.299
NIHSS scores	13 (11–16)	9 (8–14)	<0.001
Hematoma volume (mL)	20 (11–26)	10 (8–15)	<0.001
Blood WBC count (×10^9^/L)	7.4 (4.7–9.8)	6.4 (4.6–8.8)	0.127
Blood glucose levels (mmol/L)	10.3 (7.5–15.2)	8.7 (6.9–10.6)	0.002
Serum A20 levels (pg/mL)	300.6 (154.9–429.4)	110.3 (70.3–224.9)	<0.001

Data were presented as count (percentage), mean ± standard deviation, or median (upper-lower quartiles). The chi-square test, Fisher’s exact test, Student’s 𝑡-test, and the Mann–Whitney test were used for intergroup comparison. NIHSS, National Institutes of Health Stroke Scale; COPD, chronic obstructive pulmonary disease; IVH, intraventricular hemorrhage; SAH, subarachnoid hemorrhage; IHD, ischemic heart disease; WBC, white blood cell; SAP, systolic arterial pressure; DAP, diastolic arterial pressure; END, early neurological deterioration.

## 4 Discussion

In this study, we found a significant elevation in serum A20 levels during 14 days after ICH, and another valuable point was a close correlation between serum A20 levels and sickness severity indicated by NIHSS scores and hematoma volume. On univariate analysis, serum A20 levels were independently associated with poor prognosis, SAP, and END, and these associations were verified via multivariate analysis. Moreover, serum A20 levels owned effective predictive ability of poor prognosis, SAP, and END. Models containing serum A20 and other independent predictors of poor prognosis, SAP, and END, namely, NIHSS score and hematoma volume, exhibited efficient predictive ability, clinical validity, and stability using many statistical methods. Overall, serum A20 may be a potential prognostic biomarker of ICH.

A previous study confirmed a marked increase in serum A20 levels at admission in patients with aneurysmal subarachnoid hemorrhage compared to healthy controls (22). To the best of our knowledge, this is unclear with respect to serum A20 levels following ICH in humans. In the present study, 76 patients, who allowed for blood sample collection at several time points post ICH, were able to represent all 243 patients because these patients occupied analogous baseline data as the entire patients. Subsequently, a dynamic variation in serum A20 levels post ICH was confirmed, manifested as a prompt elevation of serum levels after ICH, with a peak value at day 3, followed by a gradual reduction until day 14. Serum A20 levels of patients exhibited a significant increase during the 14 days when compared to controls. Under physiological conditions, A20 is abundantly produced in neurons and astrocytes derived from human and animal brains (16, 17). In the state of pathological insults to cerebral tissues from ischemia, trauma, and hemorrhage, A20 expression by neurons, along with astrocytes and microglia, is evidently heightened (18–21). The blood–brain barrier is considered to be at a stage of breakdown secondary to acute ICH (28); therefore, leakage of A20 from the central nervous system may be an avenue for enhancing A20 levels in human peripheral blood with acute brain injury. Nevertheless, significantly increased expressions of A20 mRNA were observed in the peripheral blood mononuclear cells of individuals with ICH (18), suggesting that A20 in the peripheral blood may be directly generated from peripheral blood cells. Because patients with acute ICH are vulnerable to systemic reactions, typified by systemic inflammatory response syndrome (29, 30), acute stimuli such as ICH may activate A20 secretion from peripheral blood cells. Overall, blood A20 may be derived from both the peripheral and central nervous systems.

A20 functionally acts as an endogenous protective factor for defending against inflammatory injuries under some inflammation-related scenarios, encompassing acute myocardial infarction, chronic hepatitis B, kidney transplantation, and rheumatoid arthritis (31–34). Intrinsically, the protective properties of A20 lie in its inhibitory effects on inflammation in macrophages, dendritic cells, T cells, B cells, and other immune cells in the peripheral system (35). Through exogenous supplementation or silencing technique, A20 has been demonstrated to exert brain-protective functions by reducing neuroinflammation, alleviating brain edema, maintaining blood–brain barrier permeability, and subsequently relieving neurological deficits in rats subjected to ICH, ischemic stroke, traumatic brain injury, and subarachnoid hemorrhage (18–21). Clearly, the A20 essential mechanism in brain injury is to reduce the tumor necrosis factor receptor-associated factor 6/NF-κB signaling pathway (36). Naturally, A20 is established as an endogenous brain-protective factor. Hence, the incremental production of A20 from the brain or blood cells may be a compensatory cellular reaction to central or systemic damage following acute brain injury.

In a cohort of humans with aneurysmal subarachnoid hemorrhage, serum A20 levels at admission were closely correlated with severity according to the Hunt–Hess and modified Fisher scales (22). In the current study, 76 patients who had similar baseline features to all patients with ICH agreed to provide blood samples at seven consecutive time points after ICH, thus enabling the investigation of the association between serum A20 levels at different time points and severity reflected by the NIHSS score and intraparenchymal bleeding volume. Significant correlations were observed between the two severity indicators and serial serum A20 levels. These results indicated that the initial intensity of ICH may have a significant impact on serum A20 levels from admission to 14 days post ICH. This evidence supports the notion that serum A20 may be a prospective biomarker for assessing the severity of ICH.

For the outcome correlation study with serum A20 levels in ICH, a 6-month poor prognosis (mRS scores of 3–6) and SAP and END, which are closely related to post-ICH poor prognosis (7, 8), were selected as the three outcome variables. Although the onset time of SAP may coincide with vomiting or dysphagia, this does not indicate that all SAP is caused by vomiting or dysphagia (8). In other words, vomiting and dysphagia do not always lead to SAP. Thus, it is clinically significant to predict the occurrence of SAP. Antiplatelet therapy and hematoma expansion are the possible causes of END; and the increased NIHSS score is only a phenomenon of END (7). Consequently, it is clinically valuable that initial NIHSS scores and initial hematoma volume are utilized to predict END. Among the 76 patients who consented for blood collection at multiple time points, serum A20 levels at seven time points from admission to day 14 were significantly higher in patients with possible poor prognosis, SAP, or END than in those without the respective events. In terms of the predictive capability of serial serum A20 levels for these adverse events, serum A20 levels at admission did not demonstrate the disadvantages over those at other time points, thereby supporting the use of serum A20 levels at admission as a biochemical marker for predicting poor prognosis, SAP, and END following ICH. The number of patients in this study was increased to 243 for determining the predictive significance of serum A20 levels at admission under strong statistical power. Serum A20 levels in conjunction with NIHSS scores and hematoma volume, conventional determinants of adverse outcomes after ICH (5), were independently associated with poor prognosis, SAP, and END in this group of patients with ICH. Similarly, serum A20 levels at admission were independently associated with the 90-day poor prognosis and delayed cerebral ischemia in 112 patients with aneurysmal subarachnoid hemorrhages (22). Moreover, our study, because of its large sample size and blood collection at numerous time intervals, strongly supports the notion that serum A20 levels at admission may be an encouraging prognostic biomarker of ICH.

Serum A20 levels at admission efficiently discriminated the likelihood of poor prognosis, SAP, and END following ICH, with all areas under the ROC curve >0.75. Moreover, the additive effect of serum A20 levels on the NIHSS scores and hematoma volume was demonstrated using a model configuration. A model incorporating three independent predictors of poor prognosis, SAP, or END, namely, NIHSS scores, hematoma volume, and serum A20 levels, was constructed. The model exhibited good clinical stability, validity, and discrimination efficiency based on various statistical approaches. Thus, serum A20 levels at admission may have strong predictive ability for clinical outcomes of ICH.

Extrapolating from the current data, serum A20 can be recommended as a prognostic biomarker for facilitating risk stratification and aiding in making outcome prediction strategies in the scenario of ICH. However, this study is devoid of comparisons with existing biomarkers, and therefore some valuable information is not obtained with respect to whether serum A20 is the strongest prognostic biomarker of ICH. Moreover, causal relationships between serum A20, poor prognosis, SAP, and END of ICH have not been assessed in the present study. As a consequence, exploration of intricate interplay between serum A20 and these outcome variables and comparison of its prognostic validity with other existing biomarkers should be considered as the two research directions.

This study has several strengths and weaknesses. The strengths are as follows: (1) to the best of our knowledge, this is the first study to reveal longitudinal alterations in serum A20 levels following acute ICH. Therefore, a significant elevation in serum A20 levels was observed after ICH, with peak levels at day 3 and higher levels, relative to those of controls, persisting until day 14 post ICH. (2) Three outcome variables, poor prognosis, SAP, and END, were identified as dependent factors for statistical analysis of their association with serum A20 levels in patients with acute ICH, thereby reinforcing the notion that serum A20 may be a valuable prognostic biomarker in patients with ICH. The weaknesses are as follows: (1) In this study, individuals were diseased of supratentorial intraparenchymal hemorrhage and underwent non-operational therapies because of relatively low hematoma amount at a median value of 11 mL. Accordingly, intraventricular hemorrhage only accounted for 14.8% of all patients, and subarachnoid hemorrhage just constituted 5.8% of the whole cases. Using univariate analysis, intraventricular hemorrhage had a significantly different incidence based on the classification of poor prognosis, END, and SAP, while subarachnoid hemorrhage had no substantial distinction. On multivariate analysis, intraventricular hemorrhage was not independently associated with the preceding outcome variables. In the current study, the degree of intraventricular hemorrhage and subarachnoid hemorrhage was not assessed. Maybe, such assessments could proffer insights to these phenomena. (2) From a statistical view, a total of 243 patients are adequate for clinical assessments in this study; however, except 121 patients with poor prognosis, numbers of END and SAP, that is 74 and 60, are comparatively small, so Bonferroni correction was not done during multivariate analyses. Admittedly, Bonferroni correction is a good statistical approach, while this method is not regularly used in studies without a large sample size. Virtually, validation of the conclusions may be imperative in a cohort study with a larger sample size, and consequently this study may be preliminary, just being regarded as a pilot one. (3) In the current study, 76 patients permitting for blood collection at several time points post ICH held similar baseline data as the whole patients, but the 76 patients were not randomly extracted from the whole group of patients. Thus, selection bias should be existent. A random selection method is required in future to get more suitable study subjects for further clinical investigation, although it is very difficult for all patients to consent for blood collection at several time points post ICH.

In conclusion, temporal variation in serum A20 levels was demonstrated within prespecified 14 days after acute ICH, marked by peak levels on day 3 and persistently increased levels until day 14. Serum A20 levels determined at 14 days post ICH were closely associated with the initial NIHSS scores and hematoma volume. Moreover, serum A20 levels at admission were independent predictors of post-ICH SAP, END, and 6-month poor prognosis and exhibited high predictive value for these clinical outcomes. Hence, serum A20 levels may be strongly associated with ICH severity and worse clinical outcomes, reinforcing serum A20 as a valuable prognostic biomarker in ICH.

## Data Availability

The raw data supporting the conclusions of this article will be made available by the authors, without undue reservation.

## Footnotes

^1^ https://www.r-project.org
