# Supplementary material for: Association of serum A20 levels with stroke-associated pneumonia, early neurological deterioration, and poor neurological prognosis following acute supratentorial intracerebral hemorrhage: a prospective cohort study
Source: Front Neurol. 2025 Apr 24;16:1546934. doi: 10.3389/fneur.2025.1546934 (PMC12059376; doi:10.3389/fneur.2025.1546934)
Supplement: Supplementary file 1 [file Supplementary_file_1.docx]

# Supplementary Material

**Supplemental Figure 1**

Admission serum A20 levels and baseline National Institutes of Health Stroke Scale scores in 243 patients with acute intracerebral hemorrhage.

The Spearman test revealed that serum A20 levels at admission were positively correlated with the initial National Institutes of Health Stroke Scale scores of the 243 patients with acute intracerebral hemorrhage (P<0.001).

NIHSS denotes National Institutes of Health Stroke Scale.

**Supplemental Figure 2**

Admission serum A20 levels and baseline bleeding size among 243 individuals with acute intracerebral hemorrhage.

Using the Spearman test, admission serum A20 levels were confirmed to have a significant positive correlation with baseline hematoma size following intracerebral hemorrhage in 243 patients (P<0.001).

**Supplemental Figure 3**

Discrimination ability of serum A20 levels on the risk of poor prognosis 6 months post-acute intracerebral hemorrhage in 243 patients.

Regarding receiver operating characteristic curve analysis, the likelihood of poor prognosis was effectively distinguished by admission serum A20 levels. In addition, the Youden approach was used to identify the appropriate serum A20 level for prognostic prediction.

AUC denotes area under the curve; 95% CI, 95% confidence interval.

**Supplemental Figure 4**

Nomogram reflecting a combination model of 6-month poor prognosis after acute intracerebral hemorrhage among 243 patients.

The model incorporating three indicators–the National Institutes of Health Stroke Scale, hematoma volume, and serum A20 levels–are pictorially delineated using a nomogram.

NIHSS denotes National Institutes of Health Stroke Scale.

**Supplemental Figure 5**

Calibration curve displaying model stability of poor 6-month prognosis after acute intracerebral hemorrhage in 243 patients.

Three indices, namely the National Institutes of Health Stroke Scale, hematoma volume, and serum A20 levels, were integrated to form a model. The model exhibited satisfactory stability in a calibration curve analysis.

**Supplemental Figure 6**

Decision curve exhibiting model validity of 6-month poor prognosis after acute intracerebral hemorrhage in 243 patients.

Three metrics, the National Institutes of Health Stroke Scale, hematoma volume, and serum A20 levels, were integrated to develop a model. The model was clinically beneficial when applying a decision curve assessment.

NIHSS means National Institutes of Health Stroke Scale. Model 1 included the National Institutes of Health Stroke Scale score and the hematoma volume. Model 2 included the National Institutes of Health Stroke Scale score, hematoma volume, and serum A20 levels.

**Supplemental Figure 7**

Discrimination ability of serum A20 levels for predicting stroke-associated pneumonia after acute intracerebral hemorrhage in 243 patients.

In the receiver operating characteristic curve analysis, the likelihood of stroke-associated pneumonia was efficiently differentiated by admission serum A20 levels. Additionally, using the Youden method, an applicable threshold for serum A20 levels was selected to predict stroke-associated pneumonia.

AUC means area under the curve; 95% CI, 95% confidence interval.

**Supplemental Figure 8**

Nomogram describing a combination model of stroke-associated pneumonia after acute intracerebral hemorrhage in 243 patients.

The model incorporating three indicators–the National Institutes of Health Stroke Scale, hematoma volume, and serum A20 levels–was graphically delineated using a nomogram.

NIHSS stands for National Institutes of Health Stroke Scale; SAP, stroke-associated pneumonia.

**Supplemental Figure 9**

Calibration curve exhibiting the model stability of stroke-associated pneumonia following acute intracerebral hemorrhage in 243 patients.

Three indices, namely the National Institutes of Health Stroke Scale, hematoma volume, and serum A20 levels, were combined to develop a model. The model exhibited satisfactory stability under the calibration curve.

SAP indicates stroke-associated pneumonia.

**Supplemental Figure 10**

Decision curve assessing the clinical effect of the stroke-associated pneumonia model after acute intracerebral hemorrhage in 243 patients.

Three metrics, the National Institutes of Health Stroke Scale, hematoma volume, and serum A20 levels, were merged to develop a model. The model was clinically validated by utilizing decision curve analysis.

NIHSS signifies National Institutes of Health Stroke Scale. Model 1 included the National Institutes of Health Stroke Scale score and the hematoma volume. Model 2 included the National Institutes of Health Stroke Scale score, hematoma volume, and serum A20 levels.

**Supplemental Figure 11**

Distinguishing the effect of serum A20 levels on the risk of early neurological deterioration after acute intracerebral hemorrhage in 243 patients.

Based on the receiver operating characteristic curve analysis, the likelihood of early neurological deterioration was well discriminated by admission serum A20 levels. In addition, using the Youden method, an optimal criterion for serum A20 level was identified to predict early neurological deterioration.

AUC indicates area under the curve; 95% CI, 95% confidence interval.

**Supplemental Figure 12**

Nomogram outlining the combination model of early neurological deterioration after acute intracerebral hemorrhage in 243 patients.

The model containing three scaling variables, the National Institutes of Health Stroke Scale, hematoma volume, and serum A20 levels, was visualized using a nomogram.

NIHSS denotes National Institutes of Health Stroke Scale; END, early neurological deterioration.

**Supplemental Figure 13**

Calibration curve exhibiting model steadiness of early neurological deterioration following acute intracerebral hemorrhage in 243 patients.

Three parameters, National Institutes of Health Stroke Scale, hematoma volume, and serum A20 levels, were combined to establish the model. The model exhibited good stability within the framework of the calibration curve approach.

END indicates early neurological deterioration.

**Supplemental Figure 14**

Decision curve evaluating the clinical effectiveness of an early neurological deterioration model following acute intracerebral hemorrhage in 243 patients.

A model was built by integrating the National Institutes of Health Stroke Scale score, hematoma volume, and serum A20 level to predict early neurological deterioration. The model was clinically beneficial based on the decision curve analysis.

The NIHSS stands for the National Institutes of Health Stroke Scale. Model 1 included the National Institutes of Health Stroke Scale score and the hematoma volume. Model 2 included the National Institutes of Health Stroke Scale score, hematoma volume, and serum A20 levels.


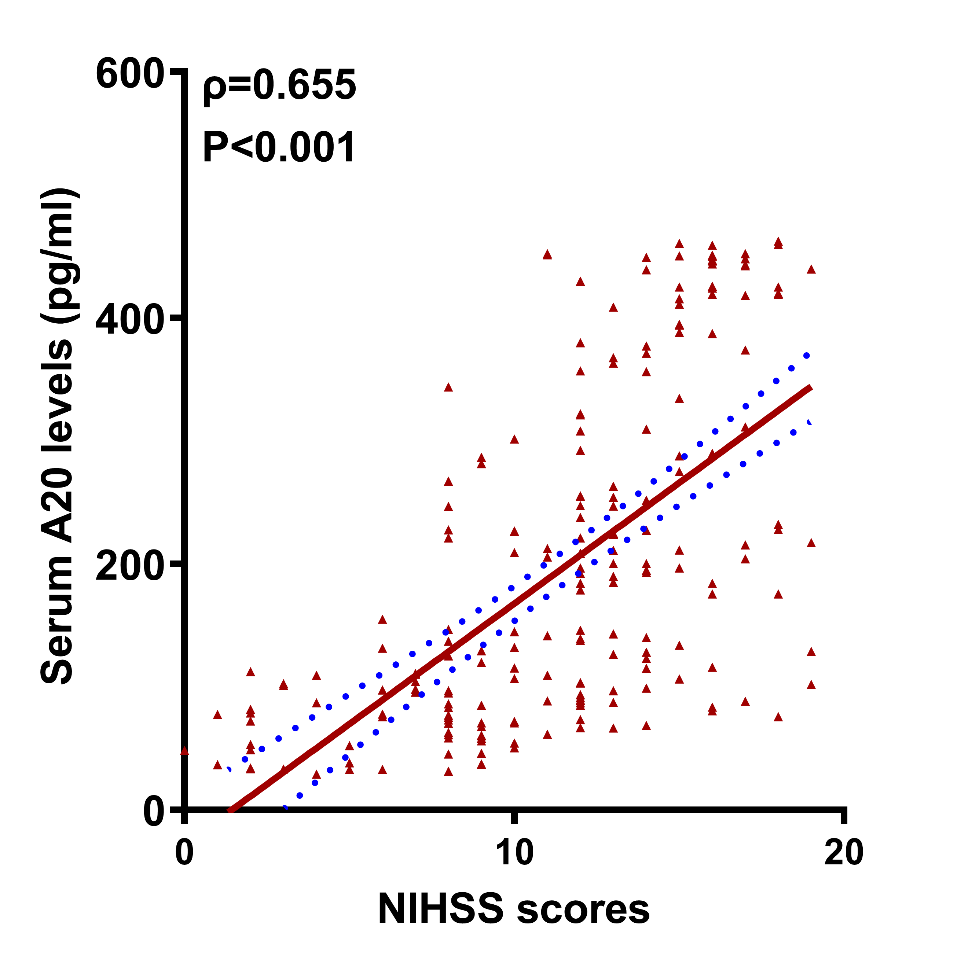


**Supplemental Figure 1**


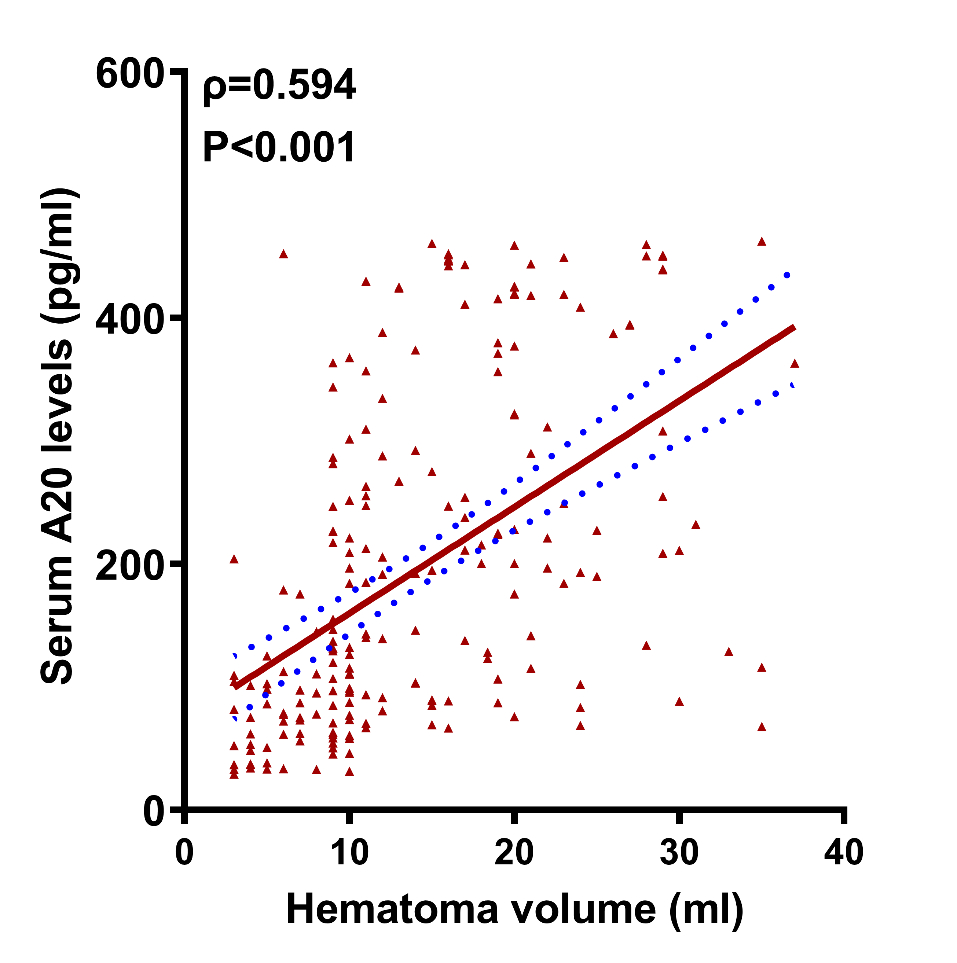


**Supplemental Figure 2**


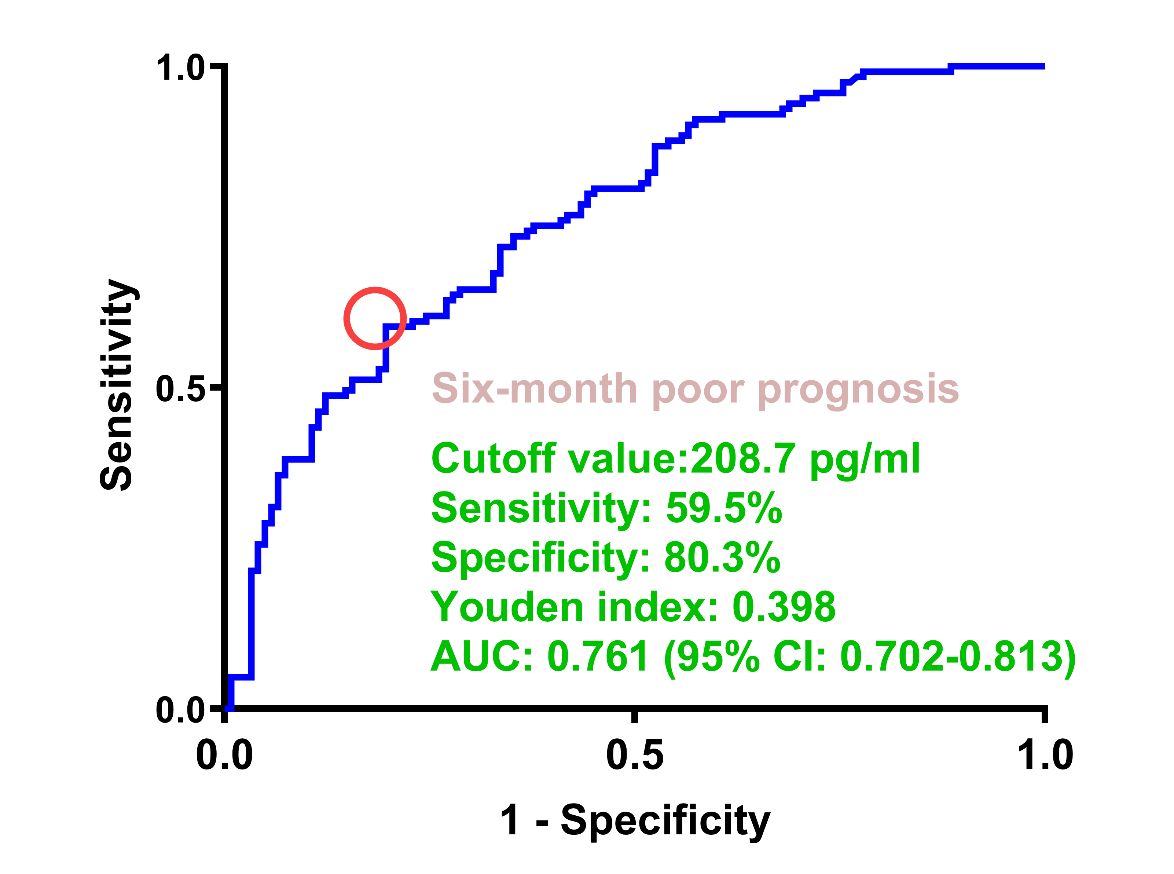


**Supplemental Figure 3**


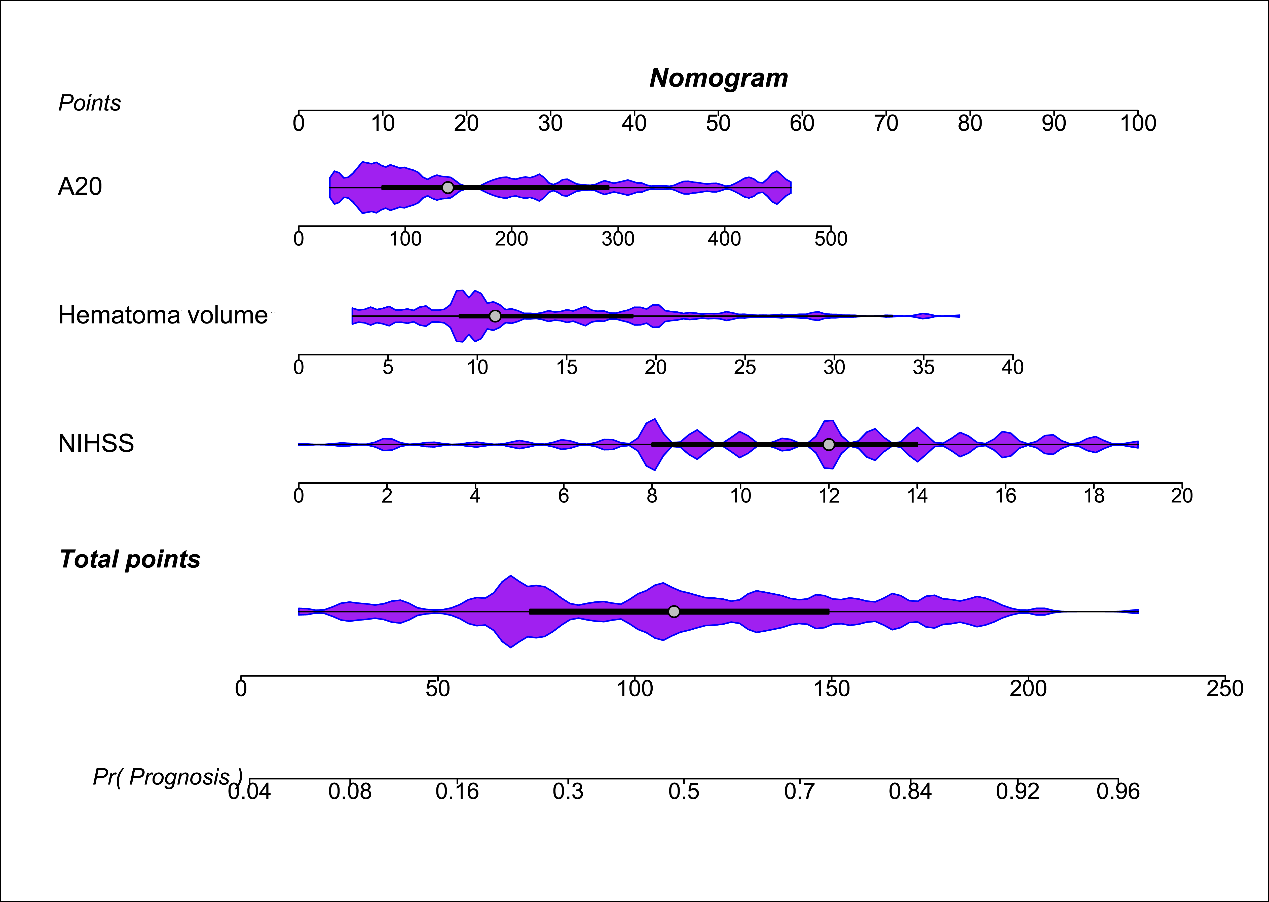


**Supplemental Figure 4**


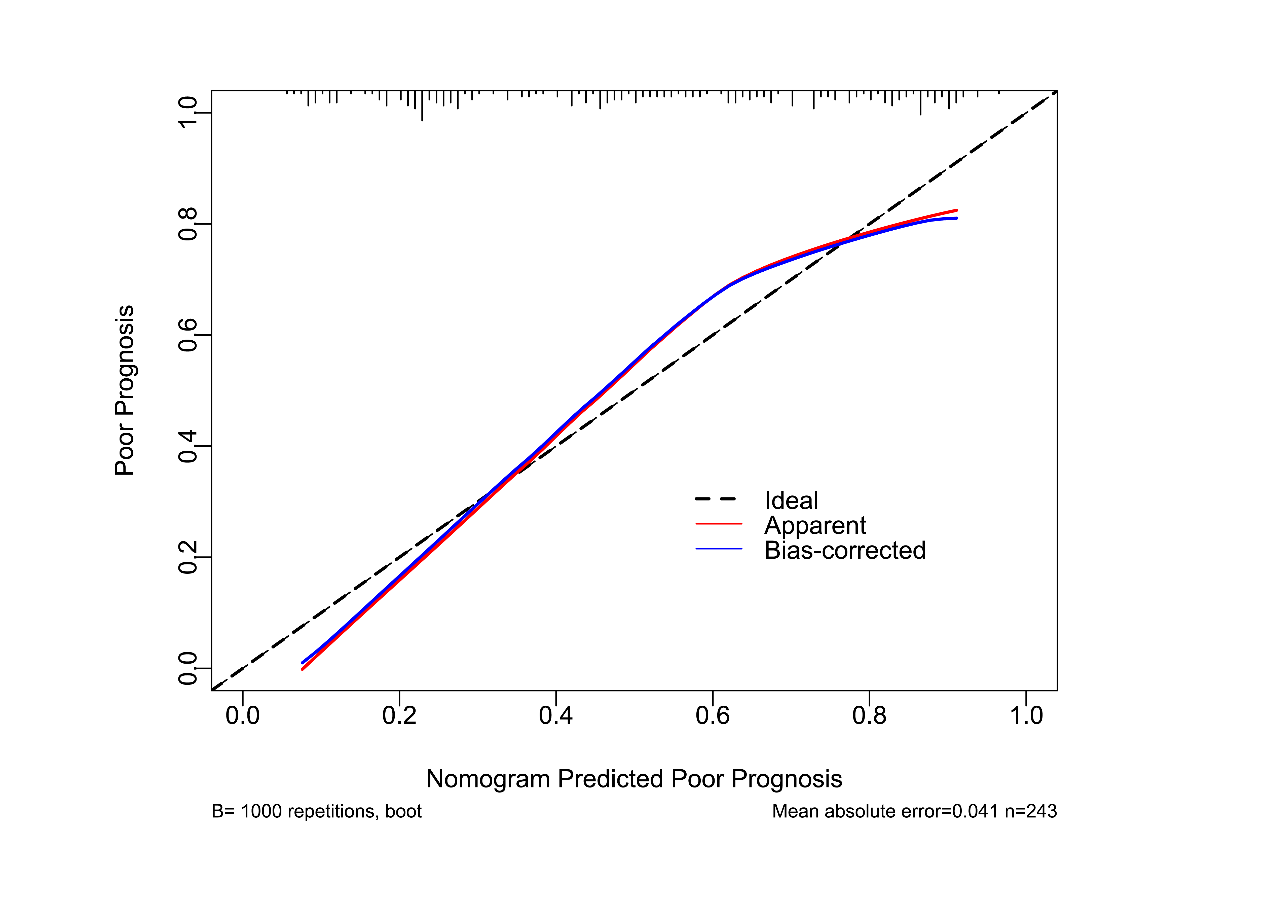


**Supplemental Figure 5**


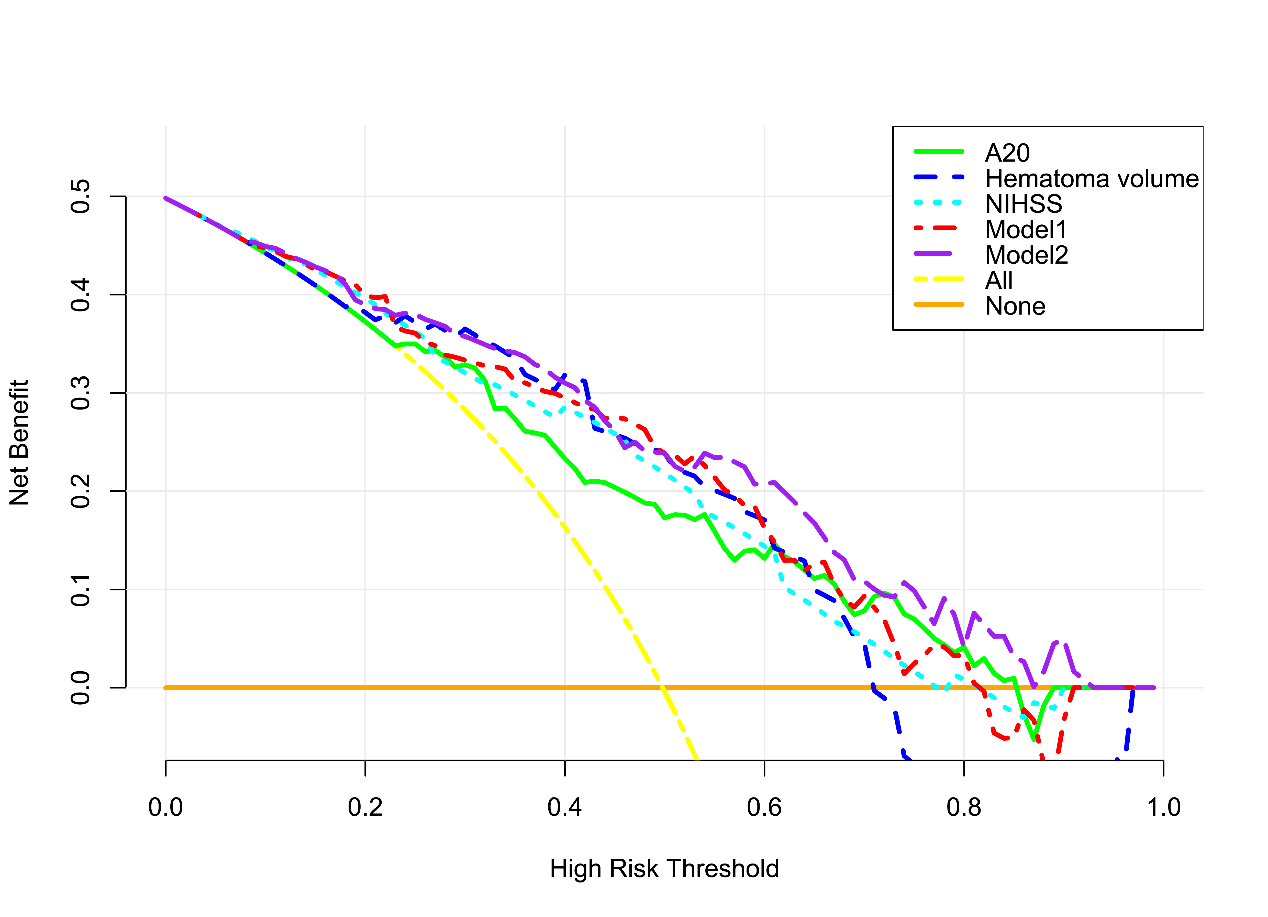


**Supplemental Figure 6**


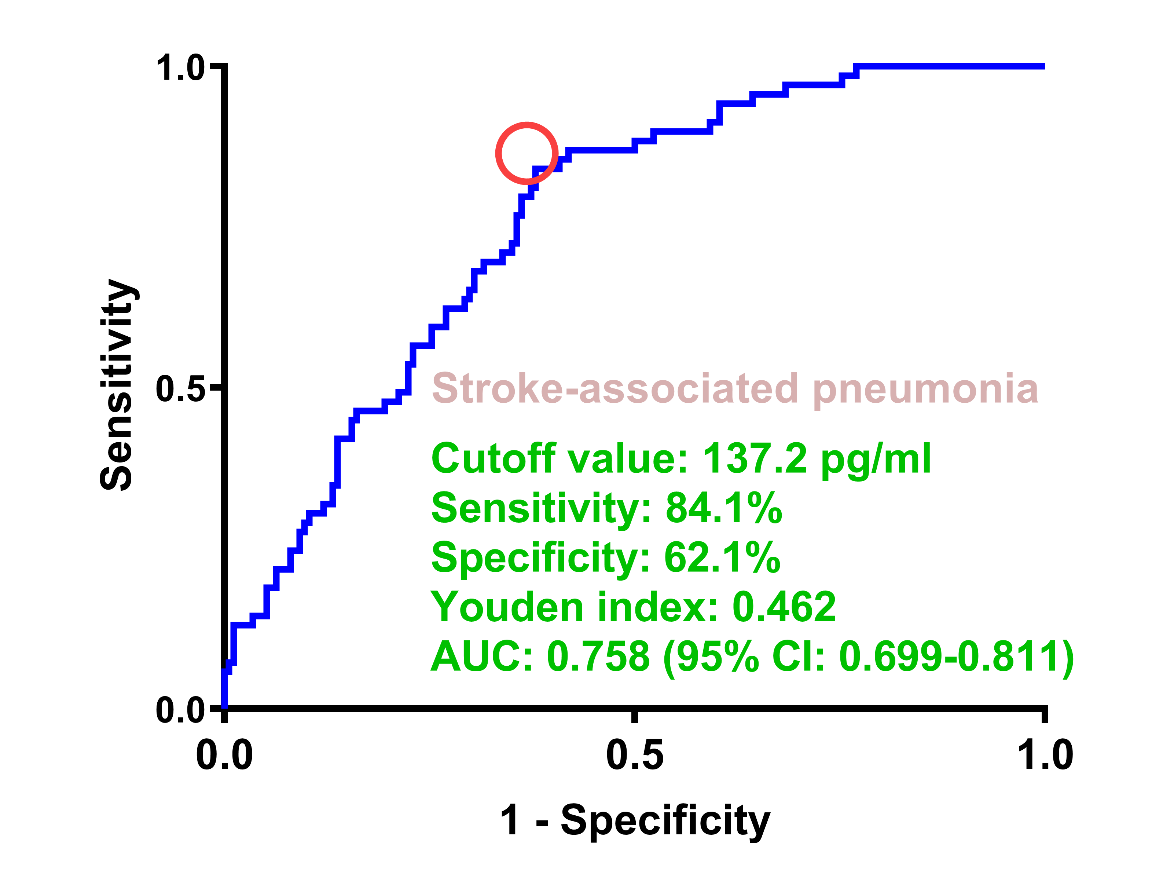


**Supplemental Figure 7**


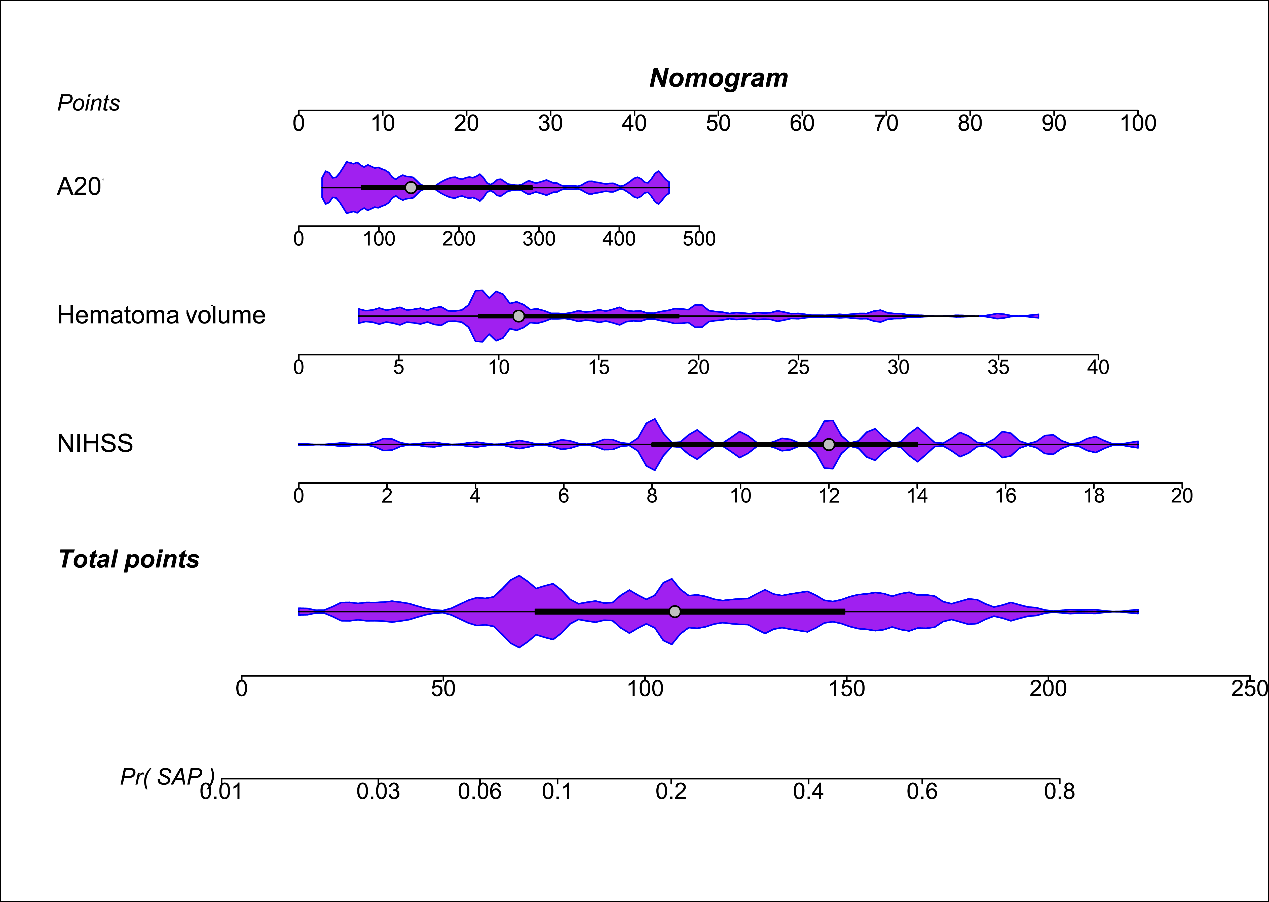


**Supplemental Figure 8**


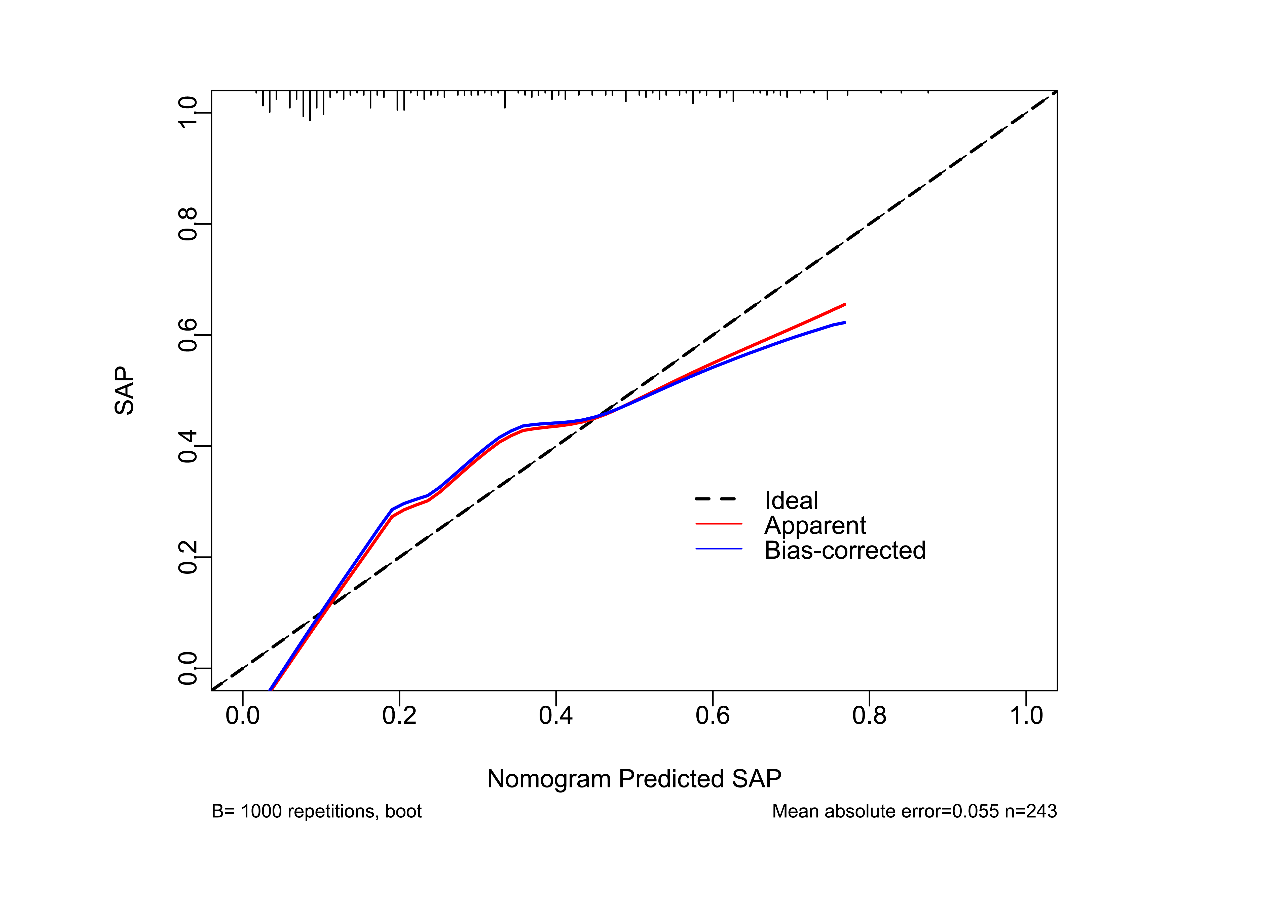


**Supplemental Figure 9**


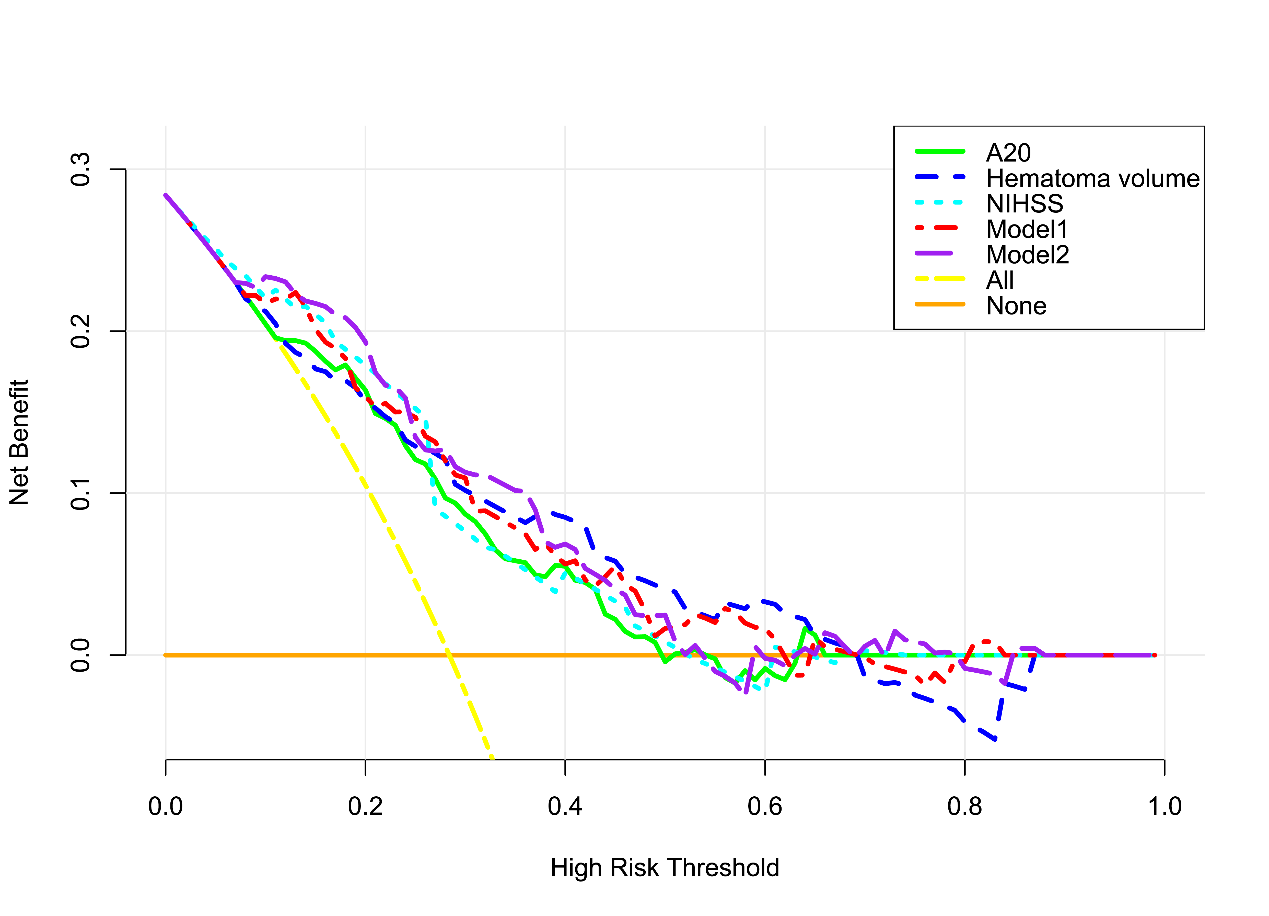


**Supplemental Figure 10**


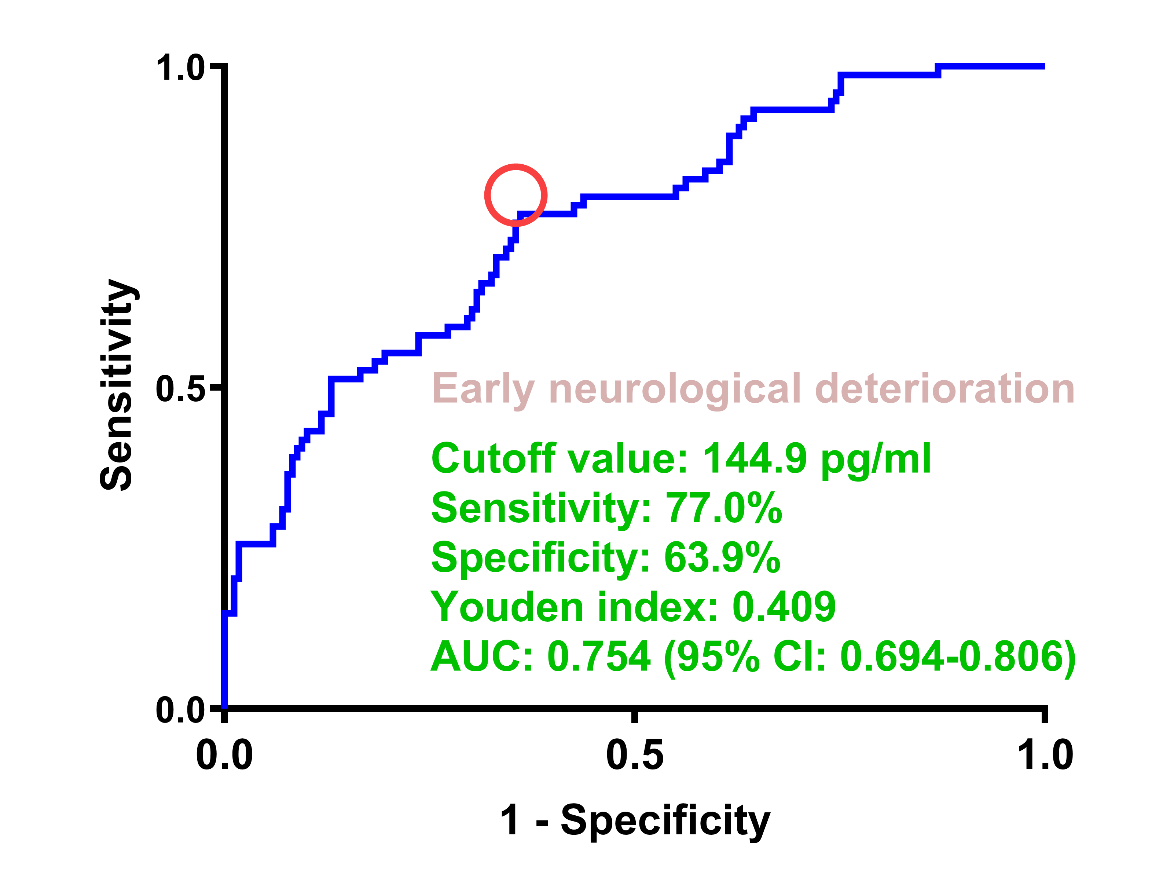


**Supplemental Figure 11**


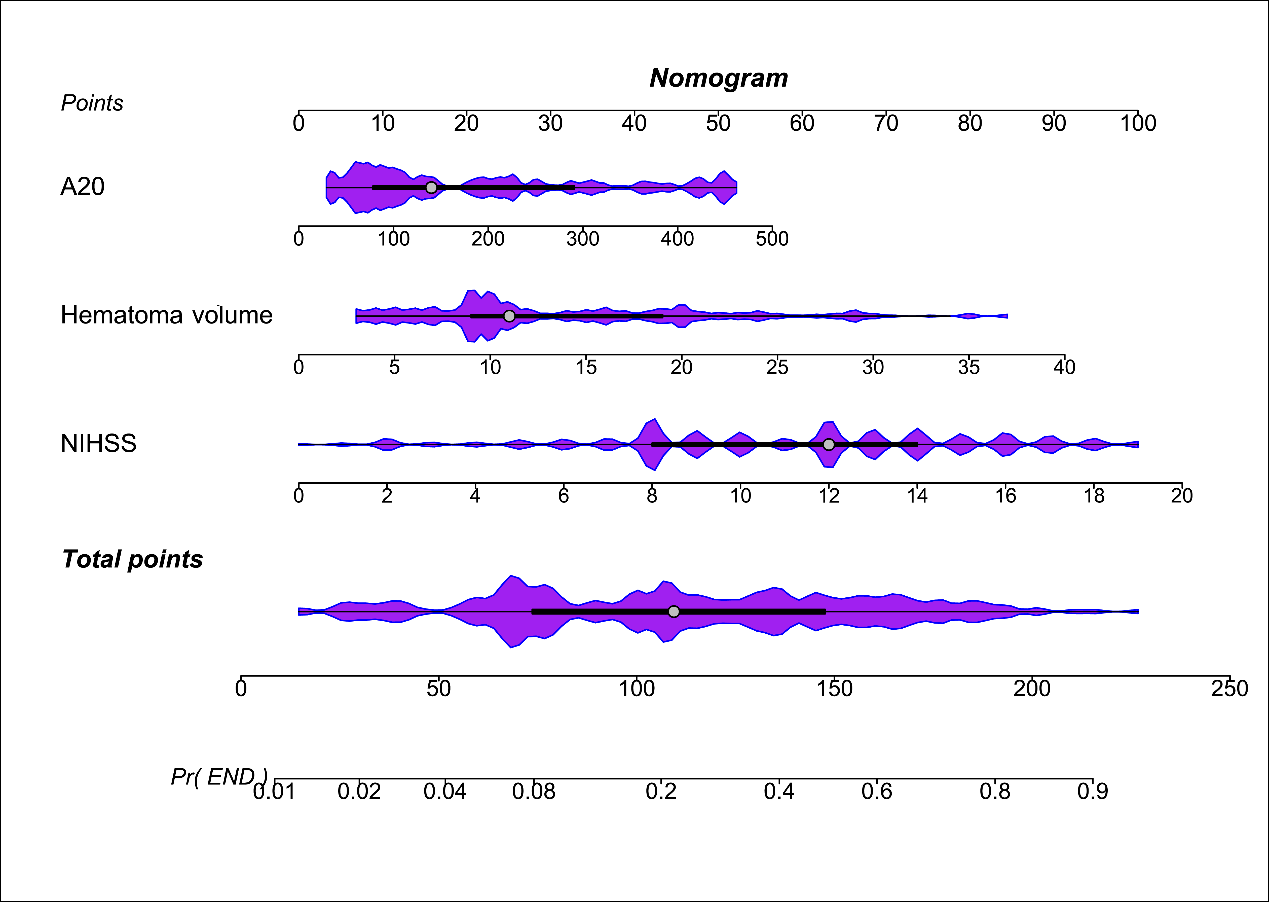


**Supplemental Figure 12**


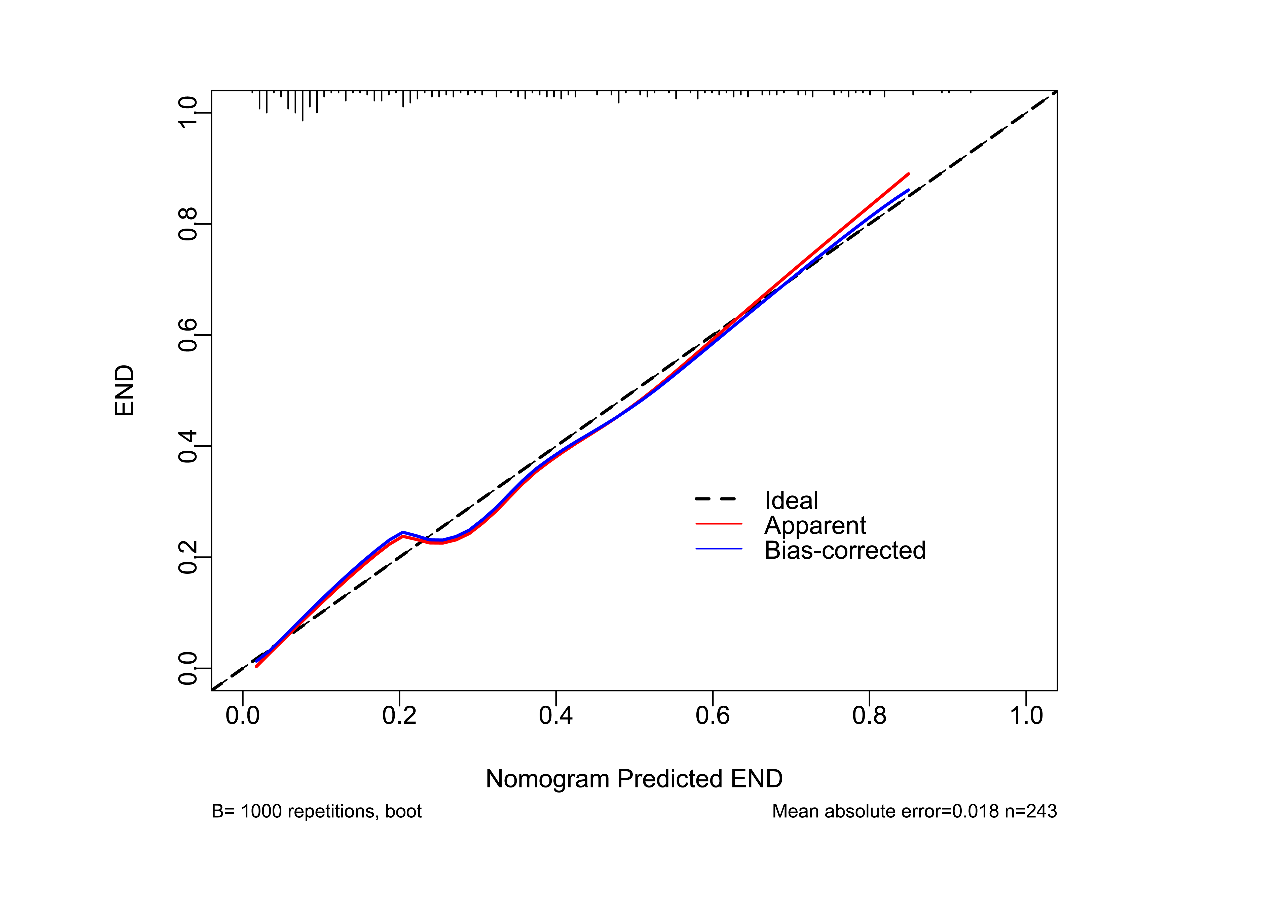


**Supplemental Figure 13**


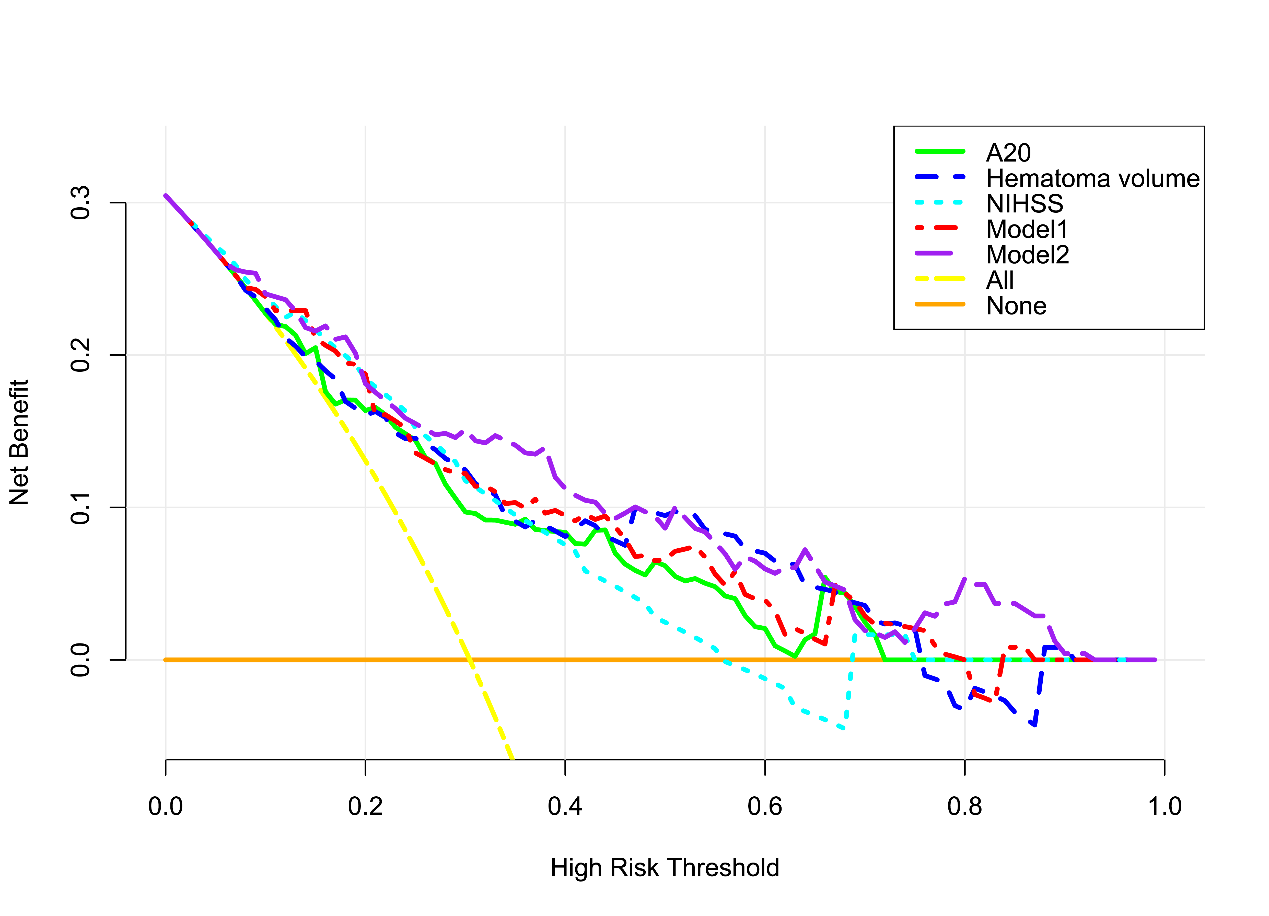


**Supplemental Figure 14**
